# Host cell protein impurities in therapeutic proteins: overview of advances in detection, nonconventional removal technologies and immunogenicity assessment

**DOI:** 10.1186/s13036-026-00685-2

**Published:** 2026-05-19

**Authors:** Sakhr Alhuthali, Syazana Mohamad Pauzi, Cleo Kontoravdi

**Affiliations:** 1 Chemical and Materials Engineering Department, Faculty of Engineering, King Abdulaziz University, Jeddah, 21589 Saudi Arabia; 2 Department of Chemical Engineering, Imperial College London, London, SW7 2AZ UK

**Keywords:** Host cell proteins, Chinese hamster ovary cells, Therapeutic proteins, Monoclonal antibodies, Critical quality attributes, Cell-derived impurities

## Abstract

Therapeutic proteins, particularly monoclonal antibodies, represent a cutting-edge technology for combating chronic illnesses. These biomolecules are produced in cell-based expression systems that generate thousands of impurities, which must be removed through a sequence of chromatographic and filtration steps to ensure drug efficacy and patient safety. Host cell proteins (HCPs) are among the most concerning impurities, due to quantification challenges, their physicochemical diversity, and their potential to affect drug stability and increase immunogenicity. Tracking and removing HCPs remains a perpetual industrial goal and a growing topic of discussion in the biotechnology industry and amongst regulators. In this review, we provide a comprehensive overview of the research about HCPs in biopharmaceutical processes, highlighting gaps in process analytics, process design, and impurity risk assessment. We summarise the challenges and recent progress in HCP detection and quantification, with extensive focus on LC-MS workflows. We next examine methods to reduce HCPs in the upstream process, followed by an overview of emerging purification technologies. Finally, we review some experimental and computational tools for predicting product immunogenicity and optimising manufacturing processes. This review focuses on advances made in the past five years and is intended to support decision-making in therapeutic protein manufacturing.

## Introduction

Biopharmaceuticals are among the most complex and expensive classes of medicines and are widely used in disease prevention, diagnosis, and treatment [1, 2]. Monoclonal antibodies (mAbs) and hormones such as human growth hormone and insulin dominate the market [3]. mAbs are commonly prescribed for cancer and autoimmune disorders, being the most promising biologic since their first approval in 1985 [4]. From 2018 to 2022, in the US and/or Europe, 197 biopharmaceuticals were approved, and the global biopharmaceuticals market size is projected to reach more than USD 500 billion by 2030, according to Global Market Insights, with 13 to 17% of that expected to come from biosimilar sales [3, 5]. In 2023, the U.S. Food and Drug Administration (FDA) approved a record 17 biopharmaceutical drugs, including two bispecific mAbs [6]. In 2025, China accounted for 10 of the 19 antibody therapeutics approved globally and currently has 65% of those under regulatory review [7]. These antibody therapeutics can be monitored annually in the Antibodies to Watch article series and on the Antibody Society webpage (http://www.antibodysociety.org) [7, 8]. A wide range of microbial, plant, and mammalian cells are used to produce recombinant proteins and peptides for therapeutic purposes [9]. These cells differ in their growth rates, post-translation modifications, and host cell proteins (HCPs) abundance and species [10–12].

HCPs are produced by viable cells and released by the lysis of dead cells during cell culture [13, 14]. HCPs are typically involved in essential cellular processes, including protein translation and folding, glucose and lipid metabolism, and stress response [13, 15, 16]. HCPs are the predominant class of impurities in biopharmaceutical cell culture systems and pose a concern for biopharmaceutical companies as they could affect product quality and might be immunogenic [17]. Several classifications of HCPs have been highlighted in the literature, including problematic HCPs that contribute to product or excipient degradation [18, 19]. Others are classified as difficult-to-remove HCPs, as they persist through downstream purification and co-elute with the therapeutic protein in polishing chromatography columns [20–22]. A final group of HCPs is classified based on immunogenicity, which has attracted increasing attention in recent years to understand their interaction mechanism with the immune system [23–25]. More effort has been devoted to detecting and removing high-risk HCPs, as they compromise product efficacy and patient safety [17, 26–28].

A standard threshold for total HCP concentration in the literature is to be below 100 parts per million (ppm) which is equivalent to ng of HCP per mg of mAb or 0.01% in the final formulated product [26, 29, 30]. Many biopharmaceutical companies use 1–100 ng/mg as a target for process development [31–33]. However, regulatory agencies such as FDA, the European Medicines Agency (EMA) and International Council for Harmonisation (ICH) do not specify a threshold for HCP concentration; instead, they require manufacturers to reduce HCPs to the lowest possible levels, as determined by a highly sensitive analytical method (The FDA recommends quantification methods outlined in the United States Pharmacopeia (USP) ⟨1132.1⟩on MS method) [34–36]. Regulatory authorities conduct case-by-case assessments of purification processes and risk reviews to determine acceptable residual HCP levels [33, 37]. Regulatory authorities consider multiple factors simultaneously, including the maximum dose, route of administration, injection frequency, and preclinical and clinical data [37]. Various measurement tools and methods are currently used for HCP identification and quantification, as sensitivity, specificity, and coverage differ across sample preparation methods, detection tools, and equipment [18, 38]. The implementation of different analytical tools, such as the gold standard Enzyme-Linked Immunosorbent Assay (ELISA), liquid chromatography and mass spectrometry (LC-MS), and computational-based assessment, provides a universal strategy on how to study residual HCPs for biotherapeutics production and other cellular products, such as gene therapy [39–42].

HCPs differ in their physical properties such as molecular weight (MW), isoelectric point (pI) and, hydrophobicity; hence, multiple unit operations with different configurations are industrially used [17]. protein A affinity chromatography column remains the most efficient unit operation for removing HCPs from mAb preparations [43]. The cost of protein A ligand (i.e., approximately 15,000 USD/L depending on scale, grade, and supplier) is almost ten times higher than that of conventional chromatography ligand and high pH washing conditions may lead to loss mAb products and reduce stability during cleaning; however, recent improvements in protein A allowed better operations during alkaline conditions [44, 45]. Currently, there is a growing trend toward identifying protein A column alternatives and strategies to extend their use to improve process economics [45, 46]. The reduction of the cost of goods through the use of innovative technologies is crucial for the marketing success of biologics, including biosimilars [1, 47]. Biosimilars’ cost of goods plays a more dominant role in pricing compared to the reference product, and their potential is particularly high in low- and middle-income countries [48].

High levels of anti-HCP antibodies detected in patients during two Phase III trials of Inspiration’s IB1001 led to the suspension of the trials, and these cases are frequently cited as examples of the adverse effects of HCPs and their avoidable negative economic impact on companies [12, 49]. Vanderlaan et al. (2018) [12] outlined several industrial cases in which persistent HCPs contributed to immunogenicity or unexpected clinical outcomes. These examples include adjuvant effects from *Escherichia coli* (*E. coli*) proteins [50, 51], co-purification of host proteins such as plasminogen activator [12, 52], TLR5 stimulation by flagellin [53], and various non-specific HCP–mAb associations including PLBL2, Clusterin, MCP-1, and TGF-β1 [54–58]. More studies have been conducted recently to understand the immunogenicity difference between a wide range of HCPs [24, 59, 60].

Cost-effective therapeutics development requires a thorough assessment of HCP immunogenicity, which is best achieved by combining in silico and in vitro evaluation during early development and preclinical stages [60, 61]. A recent industrial survey involving 19 companies revealed standard tools used for HCPs’ immunogenicity risk assessment, with CD4 cell activation and T cell: Dendritic cell (DC) co-culture being the most prevalent assays [62]. Health authorities are promoting the use of combined approaches, including predictive mathematical tools, in drug development. However, limited detection methods and standards hinder early assessment of immunogenic risk, which is crucial to prevent late-stage failure of a drug candidate [63].

**Fig. 1:**
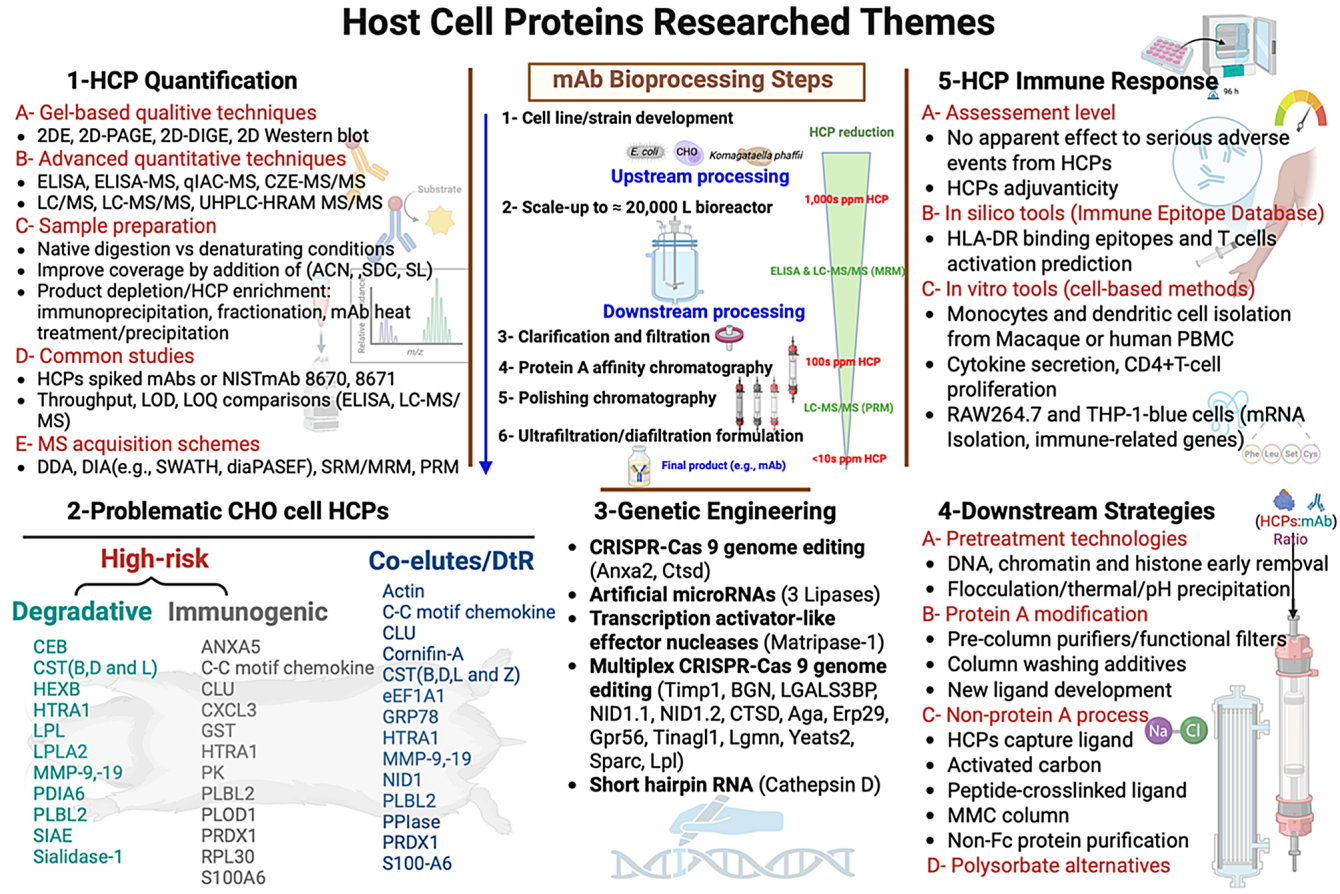
The conveyed HCP research themes discussed in this review article. Each of these five sections is discussed in the article separately. The upper-middle section shows the simplified steps of the most established therapeutic protein modality: monoclonal antibody

In this review, we summarise recent studies on five challenges associated with HCPs from common expression systems mostly over the past five years as seen in Fig. 1: (1) HCP detection and quantification approaches, (2) reported HCP species and cases of high concern from Chinese hamster ovary (CHO) and *E. coli* cell lines, (3) strategies for HCP removal during cell line development, (4) downstream HCP removal strategies including protein A column alternatives, innovative and emerging technologies to extend the chromatography column lifetime, and (5) the potential of in silico approaches to enhance our understanding of HCP immunogenic risk, along with current in vitro methods and their limitations. The knowledge condensed in this review contributes to a deeper understanding of HCP behaviour and outlines key research and analytical challenges for improved process design in biotherapeutics production, including biosimilars and next-generation therapeutics.

## HCP quantification progress and challenges

Regular analytical testing is essential throughout the drug substance and drug product life cycle, as it guides decision-making and helps prevent adverse effects. Detection and quantification of HCPs are vital for comparing the secretory machinery of different cell lines and for tracking HCP dynamics along the downstream purification train [64, 65]. Accurate measurement of HCPs is essential to enhance their removal, not only in biologics but also in drugs that may use crude enzymes during synthesis [66]. Effective removal of HCPs during therapeutic preparations is considered an essential critical quality attribute [13]. This section summarises recent advances in HCP detection and quantification, from basic gel-based techniques to advanced LC–MS workflows.

### Visual protein profiling methods

HCP species can be visually detected and quantified by two-dimensional polyacrylamide gel electrophoresis (2D-PAGE), which separates proteins by size and charge. 2D-PAGE methods use staining dyes such as fluorescent SYPRO Ruby and colorimetric silver staining, of which the former is more reliable, sensitive, and easier to use in HCP-based applications [67]. An enhanced form of 2D-PAGE is two-dimensional difference in-gel electrophoresis (2D-DIGE), which stains samples with up to three spectrally distinct fluorescent cyanine dyes before separation [68, 69]. It was reported that for a sample containing no therapeutic product, such as the supernatant from a null strain (NS), 2D-DIGE shows superior reproducibility [67]. A slightly higher level of detection can be achieved using 2D electrophoresis combined with 2D Western blot (2D-WB), a method commonly used to analyse complex protein profiles, such as CHO HCPs [70]. Capillary Western blot was recently used to assess anti-HCP antibody coverage and HCP clearance in vaccine studies, serving as an advanced version of traditional slab Western blots [71]. Optimising experimental conditions, such as reducing western blot run time, improves the visibility of low-abundance protein bands. The method was faster than ELISA for detecting low-abundance HCPs in recombinant vaccine samples.

While 2D-WB is typically used to illustrate the range of HCPs, Seisenberger et al. (2021) [68] reported that artifacts arising from the loss of conformational epitopes during protein denaturation hinder its use for evaluating anti-HCP antibody coverage. In their study, the absence of low molecular weight (MW) HCPs in 2D-WB was attributed to protein denaturation during preparation. However, other studies highlighted the lack of antibodies against low MW HCPs when developing affinity-based assays [38, 72]. Hence, a second immunisation dose during anti-HCP antibody development using a low MW fraction of the HCPs was reported to enhance antibody levels against low-MW HCPs [72]. According to the authors, this could be because smaller proteins might have fewer epitopes than their larger counterparts and are also more prone to denaturation during 2D-WB. It seems that the missed HCP spots belong to more than just low MW HCPs, as Takagi et al. (2022) [73] reported a significant difference between the CHO 34 low-recovery HCPs in comparison to 850 other CHO HCPs in their immunoreactivity profiling study using tandem mass tag (TMT) labelling. Low recovery HCPs were described as low MW, more acidic (i.e., lower isoelectric point), and less hydrophobic. Recently, Pearson et al. (2023) [71] developed an automated pseudo-dot blot integrated with capillary Western technology as a proof-of-concept to determine HCP clearance during vaccine purification from Vero and CHO cell lines. This method was reported to yield results comparable to those of commercial ELISA and to provide a shorter analysis time for measuring HCPs in recombinant protein-based vaccines. Nevertheless, further investigation is needed before it can be used with more complex, higher concentration biological samples such as mAb supernatant. The simple gel-based methods are qualitative or semi-quantitative, unlike ELISA and LC-MS methods, which are widely used in downstream process design and monitoring [42, 70].

### ELISA and its integration with other technologies

Research efforts have focused on developing methods that achieve higher HCP coverage and sensitivity using differently designed ELISA assays and diverse LC-MS workflows for in-depth analysis [74–76]. Although ELISA remains the gold standard assay for HCP quantification due to its high throughput and ease of use, it has drawbacks such as incomplete coverage, providing only global quantification rather than species-specific abundance, and being expensive and time-consuming to develop [15, 72, 77]. ELISA antibodies are perishable; hence, an immunisation campaign is required whenever needed unlike the consumables used in LC-MS sample preparation [78]. Pinto et al. (2021) [79] developed at-line colorimetric-based microfluidic chips to measure Rituximab titre, lactate dehydrogenase (LDH) and HCP for CHO cell bioreactor. They used agarose beads, biotinylated anti-CHO HCP antibodies and horseradish peroxidase (HRP)-conjugated detection antibody and HRP-labelled detection antibodies, showing results comparable to commercial ELISA kits. However, antibodies concentrations must be optimised beforehand and sample dilution is required for late-stage upstream sample. Moreover, a feedback-driven dilution strategy was suggested in their later study to improve robustness and accuracy of quantification [80]. Assay automation and high-throughput analytics are needed for biological development to improve operational efficiency and cost-effectiveness according to many reviews [81, 82].

Husson et al. (2018) [83] compared LC-MS-based HCP measurements with ELISA results and found that ELISA reported on average 8 times lower HCPs in post protein A samples. Commercial kits underestimate HCP concentrations by at least 16 times in another study [38, 66]. Incomplete ELISA coverage is inevitable due to variation in immune responses that generate anti-HCP antibodies in immunised animals during ELISA development [11]. The commercial assay does not consider variation in recombinant enzymes, chaperon proteins, and antibiotic resistance genes, as a specific pretreatment step may be needed before animal immunisation. For example, when working with plant extracts to develop an ELISA assay for plant HCPs, it is essential to deplete highly abundant proteins, such as RuBisCO, which constitutes 30–50% of total soluble protein, to enable the generation of antibodies against less abundant target proteins [84]. Even this pretreatment does not guarantee full ELISA coverage, as non-immunoreactive or weakly immunoreactive HCPs that are not detected by anti-HCP antibodies [38]. One reason for the ELISA underestimation of HCP is that individual HCP levels may exceed the reagent antibody available in the ELISA [85]. Released proteases during cell culture may degrade HCPs, therefore preventing their detection by ELISA. Moreover, ELISA protein standards used to make the calibration curve do not contain the same HCP species as the process samples. This highlights the importance of developing an in-house product-and process- specific ELISA for late-stage clinical trials [86]. Many companies rely on commercially available HCP ELISAs during early development, but transition to custom HCP ELISAs in later or commercial stages [86]. These undetected HCPs by ELISA assays can be detected by the MS-based method [73, 83].

Pilely et al. (2020) [38] developed an ELISA-MS method based on immunoaffinity purification coupled to MS to determine the *E. coli* HCPs coverage using three commercially available anti-HCP antibodies (i.e., from rabbit and goat). The proposed approach demonstrated high sensitivity and reproducibility and is recommended for HCP surveillance and screening to identify the most suitable commercial ELISA during early process development. Similarly, the quantitative immunoaffinity chromatography (qIAC-MS) method was thoroughly assessed by LC-MS to determine ELISA coverage of CHO mock cell culture fluid, protein A chromatography eluates, and multiple samples from the downstream process [72]. In this method, the rabbit anti-HCP antibodies (ELISA reagent) were coupled to magnetic particles, allowing label-free quantification. In contrast, in the method of Pilely et al., the antibodies were coated onto 96-well microtiter plates. In a third affinity-based MS study, protein G magnetic beads were used for antibody binding, as they may suppress non-specific binding compared with streptavidin-based beads [73]. It was reported that qIAC-MS is now routinely used by Boehringer Ingelheim across new product developments [72]. It is worth emphasising the different settings in each method as an example of continuous technological development and industry adoption. Although affinity purification-based LC-MS is increasingly used to evaluate anti-HCP antibodies, it faces the non-specific binding to the matrix. To overcome this challenge, the optimised version uses a conjugated cleavable linker to isolate specifically interacting HCPs, which showed higher reliability and reproducibility than other previous methods during ELISA reagent preparation [87].

### LC–MS-based approaches: significance and applications

As technological advances evolve, regulatory agencies are increasingly supporting mass spectrometry as a reliable tool for quality control in drug manufacturing [36, 88]. The suitability of each platform is determined by its analytical strengths and the bioprocessing stage in which it is applied, ranging from early discovery to lot release [89]. Some of the LC-MS workflows are given for impurity control purposes during early-stage bioprocess development, for example, detecting HCPs at 50 ppm or rapid measurement for up to 60 samples a day [90, 91]. LC-MS is versatile, extending beyond HCP detection to the study of protein aggregation and other product quality attributes. For example, Xu et al. (2021) [92] characterised subvisible particulates, identifying low levels of hydrophobic proteins prone to aggregation. Multi-attribute LC-MS approaches can simultaneously monitor HCPs, glycosylation, and product sequence variants, detecting both known and unknown peaks relative to a reference sample [64].

In standard LC–MS setups for HCP identification, a one-dimensional reversed-phase liquid chromatography (RPLC) column, typically a BEH C18 with varying lengths and pore sizes, is used [29]. Because of the similarities between proteomic and HCP bioprocess samples, many proteomics strategies have been adopted in recent years to enhance LC-MS approach for identifying HCPs [93, 94]. Although LC-MS-based HCP analysis is challenged by the large dynamic range between HCPs and the therapeutic protein, extensive sample preparation and longer LC runtimes (e.g., longer columns with nano-LC capabilities) can help achieve the required high sensitivity [29, 37, 88, 95]. Likewise, many scientists explored options in combination with the standard LC system, such as Capillary Electrophoresis (CE) [95–97]. It was shown that LC-CE-MS/MS identified twice to thrice more HCPs when compared to LC-MS/MS, but it took 35 and 22 hours per sample respectively [96].

Due to the dynamic range limitation in LC-MS, sensitivity and coverage can be improved by depleting the therapeutic protein, using a protein A column, precipitation, or a molecular cut-off membrane and by employing multidimensional LC workflows such as size-exclusion chromatography (SEC), peptides fractionation, HCP-enrichment steps, columns with charged-surface hybrid (CSH) packing [64, 70, 93, 98–102]. Injecting a higher sample load was also suggested, along with applying an exclusion list with m/z values corresponding to the high concentration proteins’ peptides, such as therapeutic product [64]. Recent reviews highlighted the importance of combining measurement tools and optimising sample preparation to expand identification to low-abundance HCPs and increase throughput [11, 26, 29]. Despite its powerful capabilities, LC-MS requires highly trained personnel, involves lengthy and complex sample preparation, and relies on expensive instrumentation [103].

#### Comparison of DDA, DIA, and targeted MS for HCP quantification

There are three primary MS detection methods, namely data-dependent acquisition (DDA), data-independent acquisition (DIA), and targeted MS [29]. DDA is commonly used for qualitative analysis and HCP monitoring, but is known to be less reproducible and biased against low-abundant proteins [104]. The Hi3 label-free method by Silva et al. [105] is widely used with DDA for protein quantification. It estimates protein amount from the average MS signal of the three most intense tryptic peptides, yielding a value comparable to that from absolute quantification using a primary reference protein. However, DIA gained popularity because it is independent of precursor ions composition during fragmentation and does not require prior knowledge, as in targeted MS analysis. Hence the DIA method offers simultaneous quantification of all detected HCPs [78]. Although building a spectral library for the DIA approach can be time-consuming, it is still faster and more cost-effective than developing a new ELISA assay, and newly identified peptides can be added during process development as long as retention-time standards are included in the samples [83]. DIA MS2 spectral libraries can be generated either experimentally or in silico using prediction algorithms (library-free approaches). Recently, a few algorithms were proposed in the literature for direct peptide identification and spectral library construction from DIA data (MSFragger-DIA and DIA-Umpire), demonstrating superior speed and accuracy compared to existing DIA analysis methods [106].

There are two types of targeted MS: selected reaction monitoring (SRM) (also called multiple reaction monitoring, MRM) and parallel reaction monitoring (PRM) [95, 104]. SRM tracks predefined precursor-fragment ion pair while PRM records all fragments of a chosen precursor and uses triple quadrupole MS and high-resolution MS, such as Orbitrap or Quadrupole time-of-flight (Q-TOF) respectively [88]. Q-TOF instrument is commonly used for global HCP profiling because it offers high-throughput capabilities in both DDA and DIA modes. The Q-TOF has lower sensitivity for detecting ultralow HCP than Orbitraps. Quadrupole-Orbitrap platform, for example, the Q Exactive and Exploris series support high-resolution acquisition methods such as PRM and BoxCar. More about emerging acquisition strategies, such as BoxCar and HCP-AIMS (i.e., analysis via iterative MS1 exclusion), can be found in the extensive MS review by Tank et al. (2026) [88]. Targeted MS analysis is used for absolute quantification of HCPs on certain occasion, primarily in a late purification-stage sample to confirm their removal, for root-cause analysis of polysorbate degradation or adverse clinical events, and for further ELISA issues, such as inconsistent readings [29].

There are some emerging techniques, such as data-dependent-independent acquisition (DDIA), which was proposed to overcome DIA in protein identification using deep learning. Although both methods yield a similar numbers of peptide identifications, DDIA provides broader protein coverage [107]. Likewise, the DIA-based sequential window acquisition of all theoretical fragment-ion spectra (SWATH) was used for absolute measurement of nine HCPs in the mAb polishing step by Carvalho et al. (2024) [85]. Field asymmetric ion mobility spectrometry (FAIMS) can be integrated with high-resolution MS platforms, particularly Q-Orbitrap and tribrid systems, to introduce gas-phase fractionation (GPF) before MS analysis [108]. Xu et al. (2025) [28] combined the DDA spectral library approach (for the CHO-K1 cell line) with MRM quantification and PRM verification to measure 28 high-risk HCPs confidently. This integrated DDA-PRM-dMRM workflow overcomes the limitation of each technique, providing improved dynamic range, specificity, and quantification sensitivity.

#### Ensuring reliability in HCP proteomics: from software tools to database selection

Quantitative HCP assessments are typically performed using tools such as Skyline and Spectronaut. Skyline is an open-source platform that offers extensive customisation but depends heavily on expert verification [109]. In contrast, Spectronaut provides a more user-friendly platform with streamlined DIA processing and preset analytical pipelines [110]. Recent advances in scoring strategies and deep-learning-based models have further strengthened peptide–spectrum matching, enabling deeper coverage and reducing false-discovery rates (FDR) [111, 112]. Most studies apply a community-standard FDR of 1–5%, typically determined using the target–decoy approach, to minimise incorrect protein identifications. However, this approach can be less reliable for small datasets, such as purified HCP samples, where FDR estimates tend to be overly optimistic. Kreimer et al. (2017) [113] highlighted these limitations and recommended using a higher FDR (e.g., 5%) in such cases. Since comprehensive HCP identification is critical for patient safety and product stability, a slightly more relaxed cutoff (5–10%), combined with manual review of potential false positives, is advisable for final drug product analysis. FDR estimation in HCPs studies is often challenged by limitations in current statistical approaches, including biases introduced by decoy-based methods and variability in how decoys are generated and evaluated [30, 114, 115].

A study published in 2019 reported 26,530 CHO genes and 47,829 unique protein sequences, highlighting the redundancy of many UniProt entries and the inadequacy of relying on a single CHO reference proteome due to its incompleteness [29, 116]. Consequently, HCP analysis can be affected by frequent updates to local UniProt databases, which often expand in size without improving data quality. Although some studies instead employ RefSeq/NCBI protein datasets, most publications still identify HCPs using UniProt accession numbers [37, 117, 118]. The limited overlap, particularly among Swiss-Prot entries, between UniProt and RefSeq further complicates cross-study comparisons. Moreover, when peptides containing point mutations are not detected, search engines cannot distinguish between corresponding protein entries and may arbitrarily assign one over the other. In such cases, both entries appear as a single protein group, reflecting database redundancies rather than genuine isoforms. The authors who extensively reviewed MS data analysis and protein identification illustrate this with the example of CHO Peroxiredoxin-1 (PRDX1) [29]. CHO Lipoprotein Lipase (LPL), a high -risk and difficult-to-remove protein, appears in Uniprot under different accession numbers with only a single amino acid difference [17, 119]. Hence, there is a need for a well-curated and accurate database, particularly in the context of benchmark HCP library for cross-comparison studies [120, 121].

#### Recent industry-related HCP LC–MS studies

In this section, we highlight the findings of recent manuscripts about HCPs detection and quantification using multiple sample preparations, protein standards, chromatography columns, MS instruments, and data acquisition modes. National Institute of Standards and Technology monoclonal antibody (NISTmAb) reference materials (RM 8670 and RM 8671) are commonly used to benchmark HCP detection accuracy and sensitivity. Sharing HCP data and removal strategies can accelerate process optimisation, but differences in LC-MS workflows between laboratories hinder consistent benchmarking. Establishing industry-wide standards would significantly improve comparability and reliability [17].

**Fig. 2:**
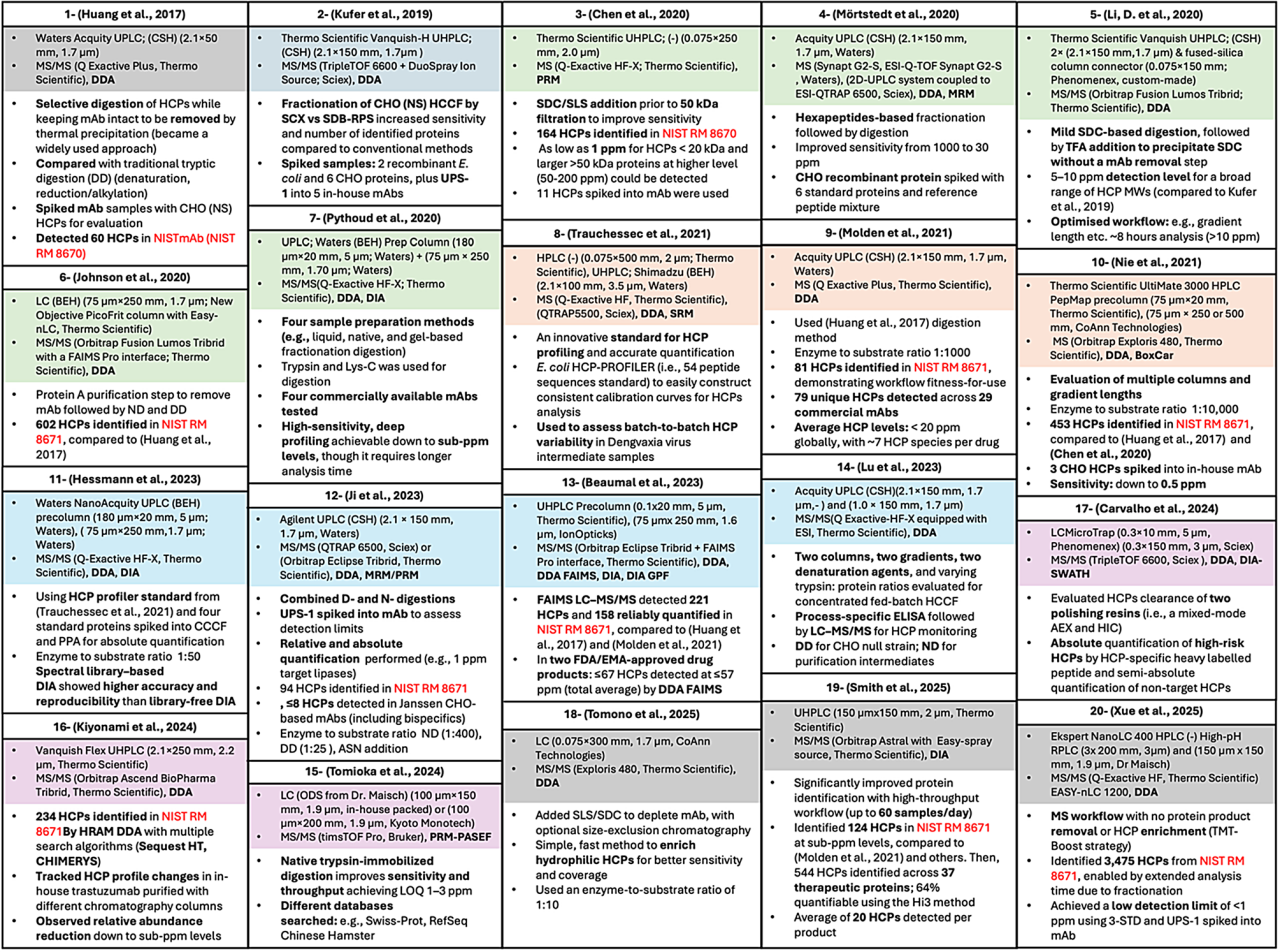
HCP detection and quantification studies published mainly in the last five years show wide variation in LC–MS workflows, including differences in protein fractionation, digestion protocols, benchmark analytes, LC column dimensions and chemistries, MS platforms, data acquisition strategies, and peptide database selection

As shown in Fig. 2, we start from top-left table to the bottom-right in chronological order (i, e., from 2017 to recent studies in 2025). Each table colour corresponds to a specific publication year. The first row of each table lists the authors and publication year, followed by the used LC and MS details and the data acquisition mode. The second row of each table highlights the main points of the manuscript in its shortest format. Findings follow Fig. 2, with related results sometimes discussed together thematically regardless of their publication dates. Huang et al. (2017) [99] developed a widely used novel sample preparation method that involves protein digestion and heat precipitation to remove undigested mAbs, leaving soluble HCPs in solution before LC-MS analysis by DDA. High-molecular-weight HCPs (i.e., >60 kDa) were detected at levels as low as 0.5 ppm, and a larger number of HCPs were identified in biopharmaceutical samples with shorter cycle times compared to traditional shotgun 2D-LC-MS.

The second study shown in Fig. 2 describes a commercial kit that combines denaturation, alkylation, and reduction into a single step before protein digestion, followed by a six-step 2D offline fractionation stage to address the dynamic range problem [93]. This method detected multiple HCPs at concentrations as low as 5 ppm and was compared with the standard protocol of Walker et al. (2017) [90] and the improved native, non-denaturing digestion of Huang et al. (2017) [99]. The native digestion workflow is simpler, faster, and suitable for relatively clean samples (e.g., bioprocess samples), whereas peptide fractionation is more time-consuming but provides superior coverage across all sample types in HCP analysis. In contrast, Chen et al. (2020) [98] used a MW cutoff membrane (i.e., 0.5 and 50 kDa), followed by centrifugation at 13,000 rpm for 8 min, to obtain antibody-depleted samples. Dissociation of HCPs-antibody interactions was achieved by addition of anionic detergents (i.e., sodium deoxycholate (SDC) and Sodium Lauroyl Sarcosinate (SLS)). This method enables the detection of low-MW HCPs (i.e., 11 kDa and 17 kDa) at concentration as low as 1 ppm. This method identified approximately 3 times more HCPs than the method developed by Huang et al. (2017) [99]. It is worth noting that Huang et al. (2017) [99] and Chen et al. (2020) [98] used the publicly unavailable NISTmAb RM 8670, whereas remaining studies in this review used the commercially available RM 8671 for benchmarking. They are produced from the same source following the same process, but the latter is a product of multiple homogenised bulk containers, as mentioned by Schiel and Turner (2018) [122]. Hence, the two standards may still differ slightly in their HCP species, which is never discussed in the literature, although they have been directly compared in many papers [91, 101, 108].

Mörtstedt et al. (2020) [123] removed a CHO-derived recombinant protein and enriched low-abundant HCPs using ProteoMiner technology, which employs highly diverse hexapeptides to fractionate proteins before tryptic digestion. They spiked proteins from various sources, rabbit, horse, bovine, and yeast, for HCP quantification. Some methods rely on a single reference protein, whereas others use an average estimate from multiple protein standards. The authors also highlighted carryover effects that were not eliminated by the 2-propanol injection wash between injected samples. None of the carryover peptides detected in the blank or protein standard samples were used for quantification. In another study, the column was washed with 90% acetonitrile (ACN) for 10 minutes and re-equilibrated in mobile phase A (typically 0.1% formic acid in water), and a control sample was analysed regularly to evaluate method variability [124]. It is worth noting that differences in washing protocols, the number and types of spiked proteins used for semi-absolute quantification, and the therapeutic products could lead to deviations between results and prevent a fair and valid comparison of detection and quantification across the studies.

Li et al. (2020) [125] developed a fast LC–MS/MS method with high sensitivity and robustness that does not need peptide fractionation or mAb removal steps. They introduced a new digestion workflow using the trypsin-compatible detergent SDC, rather than the commonly used strong denaturants such as guanidine HCl or urea, to effectively denature the mAb before enzyme digestion. The addition of ACN and acidic precipitation before and after digestion improved method robustness by increasing the recovery of hydrophobic peptides. Gradient length was assessed for a 60 $\mu$g sample load, and 177 min was chosen, representing the middle of the three explored values. The workflow reliably detected HCPs present at 10 ppm. The SDC-based method also showed similar sensitivity to that of Kufer et al. (2019) [93] for detecting 5 ppm of the 7-STDs spiked in, while requiring only one-third the sample amount. These findings emphasise how sample additives and LC operating conditions can improve sensitivity and accelerate analysis. In contrast, Johnson et al. (2020) [101] applied compensation-voltage switching using high-field asymmetric ion mobility spectroscopy (FAIMS) to enhance HCP detection. This approach identified more HCPs in the NISTmAb standard than any previously reported MS method as of 2020. Their workflow leveraged advanced MS instrument features, such as gas-phase separation (GPS).

Pythoud et al. (2021) [37] used four sample preparation protocols, including the native digestion from Huang et al. (2017) [99], and two data-acquisition methods to analyse four commercially available mAbs. HCP levels of a few tens of ppm were measured in these mAb samples, with sensitivity reaching the sub-ppm level after removing abundant mAb. As previously mentioned, fractionation-based sample preparation was ideal for deep profiling of HCP content in therapeutic samples but led to a time-consuming analytical workflow. The optimised liquid and native digestion protocols offer robust, daily applicable analyses for accurate HCP measurement, with 5 hours of preparation time for the optimised liquid digestion protocol.

Ma and Kilby (2020) [91] applied the digestion protocol of Huang et al. (2017) [99], replacing thermal precipitation with a C18 desalting spin column to enable automated workflows while maintaining comparable HCP detection. By introducing a high-pH fractionation step, they increased HCP detection to 171 species while reducing analysis time. The native digestion of Huang et al. (2017) [99] was also adopted by Molden et al. (2021) [124] in their LC-MS/MS workflow. They first applied the method to the NISTmAb standard as a benchmark for assessing the HCP profile, yielding a comparable number of identified HCPs to that of the Ma and Kilby (2020) [91] method. The results also showed good agreement in relative abundance when compared with both the method of Huang et al. (2017) [99] and the complete tryptic digest protocol of Doneanu et al. (2015) [126]. Then, Molden et al. (2021) [124] identified 79 individual HCP across 29 commercially available CHO-derived mAbs. Fewer than 7 HCPs in each mAb were detected, with a total concentration of HCPs below 100 ppm. This workflow is therefore valuable for detecting high-abundance product- or process-specific HCPs. Similarly, Ji et al. (2023) [89] analysed eight Janssen CHO-derived mAbs for HCPs detection, revealing ≤ 8 HCPs per drug product. They identified many common HCPs similar to the list given by Molden et al. (2021) [124]. Yang et al. (2022) [118] optimised native digestion of HCPs in the same NISTmAb standard, using SDC and 50% ACN prior to injection in a 500 mm nanoflow column, achieving 10 to 100 times higher sensitivity and identifying 746 HCPs, the highest number as of 2021.

Nie et al. (2021) [74] developed an ultra-low trypsin native digestion approach to preferentially digest HCPs over mAb, combined with long chromatographic gradients to enhance detection sensitivity. They extensively explored the LC design space, testing different digestion protocols, multiple column geometries, and various gradient lengths to optimise peptide separation. Similarly, Lu et al. (2023) [127] developed a long gradient LC-MS/MS method (i.e., non-denaturing digestion coupled with DDA) used in parallel with ELISA for a concentrated fed-batch mAb system (i.e., producing about 10 times higher HCP levels). This LC-MS/MS method revealed a greater number of HCPs in downstream process designed to remove high-risk HCPs.

Ji et al. (2023) [89] developed an optimised sample preparation method for the absolute quantification of lipases at the 1-ppm level, using 10% ACN to reduce non-covalent binding of HCPs to mAbs and minimise the loss of hydrophilic peptides. Quantification relied on at least two unique peptides at a 95% confidence level rather than the Hi3 approach. The method achieved a limit of detection (LOD) of 0.6 ppm, which was enhanced to 5 ppb with nano-flow column. Consistent with earlier findings, denaturing digestion detected more HCPs in complex samples such as harvested cell culture fluid (HCCF), whereas native digestion performed better for cleaner samples. Some HCPs were method-specific, supporting the use of both digestion strategies to maximise coverage. Using the NISTmAb, 94 HCPs were identified, including 78 already reported by Molden et al. (2021) [124]. Although nano-flow LC combined with HCP enrichment could further improve sensitivity, enrichment steps may lead to loss of key HCPs and reduce robustness. The optimised workflow was applied to profile HCPs in five process-intermediate samples from five CHO-derived mAbs.

Tomioka et al. (2024) [128] developed an optimised in-column native digestion workflow that significantly reduced digestion time and increased the number of quantified HCPs from 154 to 226 compared with denatured in-solution digestion. They selected the RefSeq Chinese hamster database after finding it produced more peptide identifications than TrEMBL. Using a monolithic silica column and synthetic peptides derived from high-risk HCPs, they established a high-throughput quantification method capable of quantifying high-risk HCPs (i.e., clusterin and the serine protease HTRA1) at the 1–3 ppm levels. However, comparison with earlier studies is limited because no reference mAb was used. Moreover, Tomono et al. (2025) [129] introduced the surfactant-assisted acid precipitation (SAAP) technique to enrich lower-pI and hydrophilic HCPs. The optimised protocol, 80 mM SDC, 40 mM SLS, 30-minute incubation, and 1% trifluoroacetic acid (i.e., TFA) achieved 99.9% mAb removal and was similar and less labour-intensive than immunoprecipitation depletion or chromatography-based fractionation. For less hydrophobic mAbs, adding 200 mM ammonium sulfate improved precipitation through salting-out effects, exposing hydrophobic regions and facilitating binding to the surfactant tails. Compared with other enrichment methods, SAAP serves as a complementary strategy for broader HCP coverage, and additional 97 new HCPs were detected when size exclusion chromatography (SEC) fractionation was combined with SAAP. It is worth mentioning that surfactant addition is not compatible with electrospray ionisation MS as it supress ionisation hence the addition of TFA acid was implemented in SAAP approach to precipitate SDC and SLS [129, 130].

Trauchessec et al. (2021) [131] developed the HCP-PROFILER standard, which comprises peptide sequences from 18 *E. coli* proteins, coated on a soluble biopolymer. The peptides were in different concentrations to generate an internal calibration curve for quantifying HCPs in intermediate vaccine (i.e., from Vero cells) samples during purification. Hessmann et al. (2023) [78] combined the six-point internal calibration curve from Trauchessec et al. (2021) [131] with an optimised DIA library for the detection and global measurement of CHO HCPs. The library-based DIA approach outperformed library-free DIA in accuracy, sub-ppm sensitivity, and reproducibility. Developing a comprehensive spectral library for the mAb product within a week is feasible for process development and batch-to-batch consistency. However, like ELISA, spectral-library DIA is limited to identifying only those HCPs already included in the library. Carvalho et al. (2024) [85] developed a SWATH-MS workflow that enables simultaneous detection and quantification of nine high-risk HCPs. The study provides a valuable framework for highly accurate HCP quantification to optimise the purification process.

Chrone et al. (2025) [36] assessed and validated the three quantitative LC-MS HCP methods described in USP < 1132.1>: HCP quantification relative to the product protein, a spiked-in protein, and a spiked-in peptide. Using ICH Q2(R2) criteria, they demonstrated each method’s linearity, accuracy, precision, and specificity, offering practical guidance for method selection from early development through GMP quality control. Guo et al. (2025) [110] optimised reference protein selection for SWATH-based HCP quantitation, showing that suitable standards (e.g., Bovine serum albumin and bovine carbonic anhydrase 2) enable accurate measurement of CHO PRDX1 which was validated by MRM-MS/MS. They emphasised that spiking-reference suitability depends not only on MW and pI but also on physicochemical properties of tryptic peptides and ionisation efficiency. Using this strategy, they confirmed GRP78 HCP levels below 50 ppm by SWATH-MS using both a spiked-in reference protein and a spiked-in recombinant HCP.

Kiyonami et al. (2024) [119] applied the digestion method of Huang et al. (2017) [99] in combination with ultra-high-pressure LC and high-resolution MS to evaluate HCP removal using a newly introduced mixed-mode cation-exchange chromatography for Trastuzumab. Using multiple database-search algorithms (e.g., Sequest HT and CHIMERYS) increased the number of identifiable HCPs, highlighting the benefit of multi-algorithm analysis. To improve peptide separation and sensitivity, a 250 mm LC column was used, providing higher resolving power and greater loading capacity, enabling larger sample injections and stronger MS signals.

Beaumal et al. (2023) [108] integrated FAIMS into a DIA workflow to improve selectivity and reproducibility, enabling the identification of 221 HCPs, of which 158 were quantified reliably. It is important to note that digestion enzymes vary across studies; in this case, a 1:400 Trypsin/Lys-C: protein ratio (w/w) was used, whereas others used a 1:100 trypsin: protein ratio (w/w) [94]. Despite the clear gains in HCP coverage, the added complexity and validation burden of FAIMS may limit its routine use in GMP environments. However, Smith et al. (2025) [132] used the Orbitrap Astral MS to perform sensitive, high-throughput HCP analysis, achieving a workflow of 60 samples per day without compromising protein identification. Across 37 biotherapeutics, the system detected 544 HCPs (i.e., over 80% at concentrations below 10 ppm). It demonstrated strong quantification performance, highlighting its utility for rapid, detailed assessment of low-abundance and high-risk HCPs. Magnetic bead-based SP3 sample preparation accelerated analysis compared with native digestion, although native digestion identified 50% more HCPs. Finally, Xue et al. (2025) [94] combined TMT-boosting with high-pH LC to generate eight fractions without product removal or HCPs enrichment, avoiding potential HCP loss during digestion. This approach enabled deep HCP profiling, identifying 3,475 HCPs from the NISTmAb, the highest reported at the time of publication. The workflow optimised several rarely discussed MS parameters, such as TMT signal boosting and maximum allowed ion injection time, to improve coverage and sensitivity. However, the total analysis time was not reported and is likely longer than in most previously published studies. When applied without a fractionation step, the method of Xue et al. (2025) [94] identified similar to approximately twice as many HCPs as methods by the long nanoflow column of Yang et al. (2022) [118] and ultralow tryptic digestion of Nie et al. (2021) [74]. However, despite identifying more total HCPs, approximately 25% HCPs were missed by Xue et al. (2025) [94] relative to the other two methods. This confirms the need for diverse sample-preparation workflows and LC–MS configurations to characterise the HCP spectrum in bioprocess samples.

## Non-mammalian cell-derived HCPs: current understanding and removal strategies

Microbes such as *E. coli* and *Bacillus subtilis* are the two primary bacterial hosts used for producing small, non-glycosylated proteins. Yeast species such as *Saccharomyces cerevisiae* and *Komagataella pastoris* (formerly *Pichia pastoris*) are commonly used for insulin and recombinant vaccine production because they can perform some post-translational modifications [9]. Yeast systems offer advantages such as rapid growth, genetic tractability, and established fermentation processes. Many yeast species are generally regarded as safe, and additional fungal hosts such as Aspergillus and Trichoderma species are also used. Plant-based expression systems, particularly Tobacco species like *Nicotiana benthamiana* and *Nicotiana tabacum*, are used commercially, including for a Chinese made COVID-19 vaccine and mAbs against Ebola [133]. HCPs from non-mammalian cells may contain more immunogenic epitopes [1], and higher molecular weight proteins in general have been reported to be more immunogenic than lower molecular weight ones [11]. Although studies are limited, this section summarises recent findings on bacterial, yeast, and plant expression systems.

### Microbial expression systems

Proteins from inclusion bodies are usually more concentrated and purer than secreted proteins, and peptide isolation is simpler due to their distinct size and density differences from HCPs [134]. However, da Costa et al. (2025) [135] recently compared the purification difference between a microbial-based biosimilar produced intracellularly and in the supernatant. Both product forms show no significant differences in quality, but the product obtained from HCCF contains fewer HCPs and is less susceptible to HCP variability (i.e., higher reproducibility) than the product derived from pellet lysates. These discrepancies may result from differences in the product’s molecular weight and its susceptibility to interact with HCPs. Wang et al. (2022) [66] shed light on HCP impurities in *E. coli* cell lysates expressing kinases and cGAS as a biocatalytic route for MK-1454 synthesis (i.e., a synthetic cyclic dinucleotide developed by Merck). They identified 13 HCPs in a purified sample as seen in Table 1 and assessed their immunogenicity potential. They showed how Na_2_HPO_4_ treatment improved the purification process and highlighted the need for in-house ELISA development. The purpose of the study was to analyse a few analytical tools and in silico immunogenicity prediction to improve process development and risk mitigation.

**Table 1:** Several reported problematic *E. coli* HCPs: the first 13 HCPs were at levels above 10 ppm in the MK-1454 drug prior to purification improvement [66], two co-purified HCPs with biotherapeutics and had clinical impact [136], four HCPs co-elute with hemoglobin-A2 during IMAC [137] and, in another IMAC study, one HCP (number 20) was identified among 18 co-purified HCPs [138]

Some problematic *E. coli* HCPs			
1- Single-stranded DNA binding protein	2- Superoxide dismutase (Fe)	3- Cysteine synthase	4- Alkyl hydroperoxide reductase C
5- Cold shock-like protein CspC	6- Cold shock-like protein CspA	7- Transcriptional regulatory protein CpxR IcIR family	8- 30S ribosomal protein S10
9- Trigger factor	10- Superoxide dismutase [Mn]	11- Nitrogen regulatory protein P-II	12- Adenylate kinase
13- Chaperone SurA	14- Flagellin	15- Ribose phosphate isomerase	16- Metal-binding protein ZinT
17- Bifunctional polymyxin resistance protein ArnA	18- Glutamine-fructose-6-phosphate aminotransferase	19- Acetylornithine deacetylase	20- Peptidyl-prolyl cis-trans isomerase

It was reported that *E. coli* high-abundance proteins are mainly located in the plasma membrane, while low-abundance are in the outer membrane with a total of 1,500 identified proteins [137, 139]. The high-abundance proteins do not necessarily persist during purification; for example, nearly 70% of the HCPs coelute with recombinant hemoglobin A2 in immobilised metal affinity chromatography (IMAC) belong to only 4% of total *E. coli* cell lysate proteins [137]. This highlights the ligand-HCP and product-HCP interactions, which should be assessed during process development. It is worth noting that *E. coli* BL21(DE3) and its derived cell lines are the most used strains for protein expression, as they are genetically modified to delete the lon (i.e., cytoplasmic protease) and ompT (i.e., periplasmic protease) genes [140].

In 2006, Bolanos-Garcia and Davies (2006) [138] provided an early list of 18 *E. coli* HCPs that commonly co-purify by IMAC. Most of that list reappeared in a 2020 study assessing the ligand and metal ions of IMAC [141]. The HCPs were classified into 3 groups based on their imidazole elution concentration: ≥80 mM, 55 to 80 mM of and 30 to 50 mM imidazole. A low-background strain of *E. coli* (i.e., LOBSTR) was engineered to reduce the affinity of two HCPs to Ni and Co resins, leading to much higher purity of the target protein [142]. Likewise, six HCPs genes were deleted to improve the capacity performance of diethylaminoethyl (DEAE) ion exchange column [143]. The IMAC capture step is often combined with additional purification steps to achieve the desired purity such as using nitrilotriacetic acid (NTA) and iminodiacetic acid (IDA) with different immobilised metal ions such as Zn, Cu and Ni. An extensive review of the downstream processing of insulin from *E. coli* can be found in Siew and Zhang (2021) [134].

Many proteins produced in *E. coli* have isoelectric points (pI) between 5.0 and 7.0. To remove most of *E. coli* HCPs, it is recommended to use anion-exchange (AEX) resins equilibrated with 50–100 mM Tris-HCl at pH 7.5–8.0, or to perform dialysis at pH 5.0–6.0. These approaches are often combined with low concentrations of urea or non-ionic/zwitterionic detergents to prevent co-precipitation of the target protein [46]. In contrast, *E. coli* HCP with pI values greater than 9.0 can then be separated using a cation-exchange resin equilibrated at pH 7.0–7.5. To mitigate the adverse effects of enzymes that may degrade the product, buffers often include 1–5 mM ethylenediamine tetraacetic acid (EDTA), which chelates heavy metals and inhibits specific proteases [144].

Li et al. (2018) [145] developed a heat-treatment process to remove HCPs and purify hepatitis B core protein particles from *E. coli*. This process can only be used with a thermally stable product in which thermal labile HCPs denature and precipitate at specific temperature and duration. This heat treatment cannot achieve the desired purity but instead reduces the HCPs to help extend the lifetime of hydrophobic interaction chromatography column. The temperature must be maintained within a specific range to prevent HCPs from penetrating the particles.

### Yeast and filamentous fungal systems

Comprehensive proteomic abundance studies have primarily focused on *Saccharomyces cerevisiae*, with an estimated proteome of 5,858 proteins [146]. These studies are crucial for specifying the most abundant cellular and expressed HCPs in recombinant systems to design optimal product purification steps. High-risk HCPs were removed from *Komagataella pastoris* HCCF using an adsorbent integrating a peptide ligand (i.e., PichiaGuard™), which captures HCPs while allowing mAb and single-chain variable fragment (ScFv) to flow through [147]. However, replacing the chromatography column in the downstream process is desired, as it is complex and typically the most expensive unit operation. This is true not only for CHO cell-derived mAbs but also for peptide production in yeast cells such as *Komagataella pastoris*, whose HCPs were separated using a 10 kDa membrane rather than a chromatography column, allowing the 4.1 kDa target antimicrobial peptide to pass through [148]. The peptide was then recovered by recirculating it in the retentate fraction of a subsequent 2 kDa membrane.

It is worth mentioning that the purification of mAb typically requires multiple chromatographic separations. However, in the downstream processing of fungi-based products, such as β-mannanase produced from *Komagataella pastoris* and polysaccharides from *Russula*, none or only a single chromatography column is used to remove HCPs from the products [149, 150]. Therapeutic proteins are typically administered via subcutaneous or intravenous injection, bypassing the gastrointestinal tract. Therefore, high purity achieved through rigorous purification, is essential to prevent adverse immune responses in patients [66]. Precipitation-based recovery of intracellular recombinant HBsAg expressed in *Komagataella pastoris* showed that numerous HCPs were also precipitated, suggesting that most of these proteins have pI values ≥ 4.2 [151, 152]. Moreover, the absence of endotoxins and oncogenes simplifies purification compared to bacterial expression systems [153].

### Plant expression systems

Plants provide a viable alternative for producing complex biopharmaceutical proteins that are toxic to mammalian cells or tend to form inclusion bodies, yielding a less potent product, in bacterial systems, such as anticancer viscumin [154]. Cereal crops such as wheat, barley, and maize have been explored as hosts for molecular pharming; each presents specific challenges. Wheat and barley contain glutelin, a storage protein linked to celiac disease, raising concerns about allergenicity. Maize, although a promising candidate, poses biosafety risks due to its tendency to cross-pollinate [155]. Producing a high-purity product is challenging with plant-based systems, as they generally generate more cellular debris than other expression platforms, particularly tobacco species, which are rich in secondary metabolites. Abiri et al. assessed the immunogenicity of HCPs co-purified with human serum albumin from *Oryza sativa*, which was approved for clinical trials in China [155]. They classified the plant-based protein as safe after immunogenicity testing in Sprague-Dawley rats, measuring multiple biological indicators, including cytokines and T-lymphocyte cell subsets, following injection.

It is worth mentioning that some vaccines and other smaller microbial products, for example, the Thailand-made Baiya plant-based SARS-CoV-2 vaccine, utilise a single purification step and have shown safety and high efficacy in preclinical studies [156]. Because vaccines are administrated infrequently (normally months and years apart), the risk of HCP-related adverse effects may be less of a concern than that for continuously injected biotherapeutics [29]. The EMA specifies a limit of 400 ng of HCPs per vaccine dose and it is still an important critical quality attribute [157, 158]. Despite this, rigorous monitoring is still required to ensure low HCP levels, verify patient safety, and maintain consistent manufacturing quality.

It is essential to know the origin of the impurities and the risk they pose once detected and measured. For example, β-glucans are large polysaccharides synthesised by various prokaryotic and eukaryotic organisms [159]. An extensive investigation into the presence of immunogenic β-glucan in a mAb product concluded that cellulose filters and sucrose-containing solutions were the primary sources of the contaminant [159, 160]. Implementing further downstream processing and additional washing steps resulted in a nearly tenfold decrease in β-glucan levels, bringing them in line with those observed in commercially available mAbs [160]. This issue could be resolved by changing the filter to another synthesised type to avoid β-glucans contamination. Hence, it is crucial to identify the best option in terms of removal efficiency and cost for impurity elimination, as thorough analysis is needed to mitigate risks early in the development phase and avoid detrimental commercial delays or clinical failure.

Panapitakkul et al. (2024) [156] identified the top 10 co-eluting *Nicotiana benthamiana* HCPs with Fc-fused vaccine during protein A Chromatography, which mainly belong to the photosynthesis-related and coenzyme groups. A freeze–thaw method was recently developed to remove RuBisCO and ribosomal proteins by inducing their precipitation followed by centrifugation; however, extended freezing periods should be avoided to prevent damage, and many proteins that cannot be removed by this simple strategy [133]. Additional methods include ammonium sulfate precipitation combined with citrate buffer fractionation, and pH/heat treatment followed by ultrafiltration/diafiltration [84].

## CHO cell HCP variation

Mammalian cells, particularly CHO cells, are by far the most widely used production mammalian cell system in the biopharmaceutical industry [86]. This widespread adoption is due to the extensive understanding of their growth requirements, metabolic pathways, and ability to perform complex post-translational modifications [161]. In addition, CHO cells present a low risk of contamination by human viruses, enhancing product safety, and have earned strong regulatory confidence, making them the preferred choice for therapeutic glycoprotein manufacturing. CHO and human embryonic kidney cells are used to produce complex glycosylated proteins rich in disulfide bonds, ensuring their functionality and compatibility with human systems [162]. Moreover, CHO cells are adaptable to various operating conditions and can reach high cell densities, resulting in high protein yields [163]. Recombinant proteins from mammalian cells exhibit higher bioactivity but are generally more challenging to purify due to post-translational modifications, lower expression levels, complex cell culture media, and more diverse cell-derived and process-related impurities [164, 165]. The composition and abundance of HCPs vary among different CHO-based systems, which could also be influenced by several factors such as clone selection, media composition, and cell culture operating parameters.

### Effect of bioreactor conditions on total HCP levels and species variation

Sahoo et al. (2024) [166] monitored global and 45 HCPs under different upstream conditions in a fed-batch culture to understand their release triggers. Their findings suggest that cell viability is only a partial contributor and is not always negatively correlated with the high HCP levels observed at the end of the culture. It would have been better to measure LDH to assess the extent of membrane damage in dead cells, as their data suggest continuous secretion of HCPs from viable cells. Wilson et al. (2019) [167] further identified bioreactor duration as a major factor contributing to HCP accumulation in their fed-batch experiment and reported a negative correlation between titre and purity.

However, findings from studies using different steady-state platforms and clones indicate that HCP levels may not differ significantly between perfusion and fed-batch cultures [168]. Notably, in another study using a GS CHO cell line, most HCPs exhibit similar abundance across clones in fed-batch cultures, but the HCP abundance profile is more stable in perfusion cultures [169, 170]. Nonetheless, perfusion cultures contain higher levels of enzymes that degrade polysorbate, whereas fed-batch cultures show increased levels of stress-related and protein-folding enzymes. These observations suggest that the mode of culture alone might not be the only reason for these species’ differences in HCPs, as they are also influenced by bioreactor operational conditions.

Large-scale proteomic analysis of the CHO-K1 cell line was extensively studied based on the CHO genome to improve protein characterisation accuracy [171]. Six potential cell viability marker proteins (i.e., CFL1, GPDH, LGALS1, PRDX1, TAGLN2, and MDH) have been identified in CHO-K1 cells and other industrially relevant CHO cell lines [172]. The markers detected a dead cell density increase above 1 × 10^6^ cells/mL based on spent media analysis, which could improve the bioprocessing control system. The degradation rates of these proteins were not provided, as some HCPs may be more stable in culture and thus unreliable proxy for dead cell density. It was shown that 47% of HCPs exhibit different expression levels between CHO-K1 cells producing IgG1 and their parental counterparts, with ageing cells showing reduced expression of problematic HCPs [173].

Other studies on CHO-K1 DP12 and CHO-K1 ATCC cells revealed comparable total protein and peptide numbers across different CHO cell lines, with 80% of the top 1,000 identified proteins shared among three null CHO lines [174]. In this study, 2D-PAGE analysis suggested that cell line origin plays a more significant role in shaping HCP profiles than culture conditions. Additionally, in CHO DG44 and DUKX-B11 cells producing Fc fusion proteins, the HCP species were similar regardless of whether the cell culture was operated in batch or fed-batch mode. However, the HCP final concentration was higher in the fed-batch due to its higher cell density. The product titre per HCPs was higher for DUKX-B11, highlighting the impact of the cell line on the HCPs profile. The main purpose of the study was to assess HCPs dynamics during culture and the negative effect of the HCPs accumulation on product quality [175].

It was highlighted that an increase in post-protein A HCPs occurred as the cell culture progressed from day 8 to 17 [16]. The study also presented that HCPs in an IgG1-producing CHO cell were linked to various cellular functions, including a large increase in stress response proteins and indicators of cell age in the last two days. Similarly, CHO-DP12 cells that produce IgG1 exhibited different HCP profiles depending on the culture conditions, with 33 HCP enriched in the post-protein A sample when the culture operating parameters were changed. Potentially immunogenic HCP species, such as H2B and CD8A, were more abundant when culture was carried out at a lower temperature, and species such as HYOU1 and TRAF3 were significantly increased at lower dissolved oxygen concentrations. These observations highlight the complexity of HCP dynamics and underscore the importance of optimisation-based approaches to reduce HCP levels during therapeutic protein production.

Trend-based studies linking problematic HCP secretion to bioreactor conditions or operating modes (e.g., fed-batch vs. perfusion) should be interpreted with caution. Apparent differences may be insignificant or influenced by analytical bias, as LC-MS methods preferentially detect more abundant HCPs. In addition, the presence of the therapeutic product can interfere with HCP quantification, potentially reducing sensitivity for low-abundance species. Perfusion processes often show lower product concentrations at harvest due to continuous nutrient feeding, product removal, and cell bleeding. Although overall productivity may differ. Because HCP levels are commonly reported in ppm (relative to product), perfusion may appear to have higher HCP content simply due to a lower product (i.e., the denominator of the ratio) rather than increased cellular HCP production. Therefore, protein A loading conditions should be standardised when comparing processes, as mAb-HCP interactions depend on both product and HCP concentrations and influence purification performance. Reporting HCP levels in both ppm and absolute units (e.g., mg/mL) is recommended, as they are governed by multiple process parameters such as culture duration and cell density.

### Interaction of HCPs with other molecules

Two key mechanisms contributing to HCP co-purification are the release of leachables from chromatin heteroaggregates and non-specific interactions between HCPs and mAbs [176]. Chromatin heteroaggregates form as a result of the highly reactive surfaces created by DNA and histones. Meanwhile, the non-specific interactions between HCP and mAbs result in mAb aggregates that contain HCPs, as evidenced by the higher levels of chaperones and proteins involved in the Unfolded protein Response (UPR) in the HCCF and protein A eluate [177]. Hu et al. (2022) [178] identified several common HCPs in high-molecular-weight aggregates, and their removal is essential because they are critical quality attributes. They showed that specific HCPs, such as C-C motif chemokines, tend to interact with dimers and aggregates. CHO HCPs were classified into three categories based on their physicochemical properties in the removal process according to Kornecki et al.: “the good”, “the bad”, and “the ugly”. The first HCPs are small (MW < 15 kDa) with either very low (pI < 4.75) or very high (pI > 10.0) isoelectric points, making them relatively straightforward to eliminate through acid precipitation and diafiltration. The bad HCPs are larger (MW > 15 kDa) and have pI values between 4.75–7.30 or 9.30–10.0, which makes them more difficult to remove. The ugly HCPs, with MW > 15 kDa and pI values between 7.3 and 9.3, are the most problematic, since they are especially challenging to distinguish from the target protein [179].

Protein engineering and concentrated antibodies at 30–60 mg/mL were reported by Luo et al. (2022) [43] to cause non-specific interactions. It was also noted that a high concentration of antibodies enhances the formation of antibody clusters and leads to a highly net positively charged environment, which attracts negatively charged HCPs. The concern with co-purifying HCPs is the inclusion of immunogenic proteins, such as PLBL2, that current analytical methods may overlook. It was shown that lipases such as PLBL2 and LPLA2 were only detectable when specific digestion methods were used [89]. Different digestion approaches can lead to the underestimation of PLBL2 levels, highlighting the importance of analytical method optimisation [76]. These analytical method concerns were discussed extensively in Sect. “LC–MS-based approaches: significance and applications”, as they are fundamental to all HCP studies and are a major source of discrepancies. It is worth noting that mAb aggregates can have a more substantial triggering effect on the immunogenic response when they co-aggregate with HCPs than when they are present alone [180]. It was recently shown that aggregates compete with mAbs to bind to protein A, with more aggregates found in the protein A elution tail [181]. It was encouraged to measure the aggregate mass fraction, as it might be proportional to the HCP content.

These findings highlight the importance of understanding HCP variations across CHO cell lines and culture conditions to refine purification strategies and ensure consistent product quality at large scale. While these studies help understand the optimal design space for therapeutic protein production, it is early to consider them definitive, as LC/MS methods for CHO HCPs have improved in the last few years, offering better resolution and reproducibility. LC-MS/MS was used in a recent study to screen protein A eluates of 23 Fc-based proteins to find common HCPs [182]. It was found that protein A eluates have a heterogeneous HCP mixture, which is specific to a particular product, highlighting drug-specific interaction. In addition, 10 HCPs co-elute were found with more than 50% of the 23 products, which was caused by the protein-protein interaction network. HCP concentration and species appear to be a function of the cell line, cell density, cell viability, bioreactor growth parameters, sample preparation method, and type of analytical tool [13, 22, 75, 167, 176, 183].

### High risk CHO HCPs

Among the diverse range of HCPs, some are classified as problematic due to their potential to affect the product’s quality, efficacy, and patient safety. They are commonly found in HCCF and in protein A eluate, with a wide range of isoelectric points [184]. They are typically categorised into three groups based on their potential interactions with the product and the immune system: difficult-to-remove, degradation of the product or excipient, and immunogenic, as summarised in part 2 of Fig. 1 in the introduction [17, 185]. HCPs that are grouped under the difficult-to-remove category are those that remain in the drug substance after purification steps, especially in the protein A eluate. Ideally, most impurities are expected to be removed in this step because the protein A ligand specifically binds to the mAbs. However, several studies have shown that HCPs such as chaperones and histone chromatin can co-purify with mAbs, compromising the effectiveness of the protein A capture step [186]. A subset of HCPs that share properties with the target product often co-purifies, regardless of the purification method used.

In typical downstream processing of therapeutic proteins, polysorbate 80 is added as a stabiliser to antibody formulations. The degradation of polysorbate 80 increases in oxidative stress, promoting the oxidation of methionine residues in the mAb product. Lipoprotein lipase (LPL) is one of the HCPs that is associated with the degradation of polysorbate 80 and may persist in the antibody product even after multiple purification steps, despite the low level of HCP [187]. It was reported that 20 enzymes were highlighted for their potential hydrolytic activity, explicitly targeting the degradation of polysorbate [188]. It was found that CHO sialate O-acetyl esterase (SIAE) degrades polysorbate 20 but not polysorbate 80 due to the presence of longer monoesters and higher-order esters [189]. The ester bonds in polysorbate can be enzymatically hydrolysed by some CHO HCPs, releasing free fatty acids during drug product storage to a degree where the excipient fails to adequately protect the protein, leading to particle formation or other serious immunogenic response [156, 190]. Alternatives to polysorbates (e.g., poloxamer 188) were reviewed and suggested in the literature [188].

Another concern regarding degradation is cathepsin B, a known protease that contributes to antibody fragmentation. Fragmentation of bispecific mAbs was highlighted by cathepsin, with no contribution observed from serine proteases HTRA1 and HTRA2 [191]. In addition, hexosaminidase B was reported to degrade N-glycans, which is another product quality attribute to be monitored during mAb production, although its clinical impact on patients is believed to be minimal [39]. This highlights the importance of precisely controlling bioprocess variables as each can affect multiple critical quality attributes simultaneously. Beyond these impacts, problematic HCPs can also negatively affect downstream process performance. Histone and chromatin, for instance, may bind to the protein A ligand, limiting its accessibility and reducing the efficiency of product purification [192]. This could later hinder the reusability of protein A resin, in turn, affect the cost of downstream processing.

## Recent technologies to tackle HCP generation and removal

The manufacturing process for mAbs from CHO cells is well established, and there is pressure to reduce production cost through cell line engineering, enabling less cost-intensive unit operations and process optimisation [65, 193, 194]. Protein A affinity chromatography columns account for 50% of downstream processing costs [22, 195]. Hence, multiple published studies aim to find cheaper alternatives in terms of capital and operating costs without compromising product quality [196, 197]. For example, continuous chromatography columns with real-time titre measurements increase column utilisation while minimising product loss [198]. It is worth noting that HCP removal and downstream processing accounted for the largest share of patents on biological products registered between 2020 and 2021, relative to the other 13 topics [199].

### HCP reduction in upstream process

Due to challenges posed by the problematic HCPs, efforts have been channelled towards reducing their presence early in the production process. Upstream strategies, including cell culture optimisation and targeted gene modifications, aim to lower HCP levels at the source and ease the burden on downstream processing. Cell culture optimisation, in general, involves media optimisation by manipulating the concentrations of medium components and process optimisation by evaluating critical process variables such as pH, temperature, and harvest time [14, 16, 200]. Nevertheless, the mode of cell culture operation can also contribute to reducing HCP content during upstream processing, as perfusion culture has been reported to produce a lower HCP burden compared to fed-batch culture [201]. The removal of dead cells continuously was performed using an inertial microfluidic device, which improved continuous culture performance and product purity [202]. Cell-free manufacturing platforms have gained attention in recent years for mAb-based products [203]. However, it is known that these systems alleviate some challenges in the upstream process at the expense of greater HCP removal in the downstream train, as cell lysate yields much higher HCP abundance than the therapeutic product expressed extracellularly.

Another effort to reduce HCP content in the upstream culture is through various gene modification approaches, as summarised in Fig. 1, part 3, of the introduction. These approaches aim to either reduce problematic HCPs or address the sources that lead to their expression in cell culture. Various methods have been implemented to date, including CRISPR-Cas9 genome editing [204], artificial microRNAs [205], transcription activator-like effector nucleases (TALENs) [206], and short hairpin RNA (shRNA) [183]. CRISPR-Cas9 has been regarded as a feasible approach for genome editing, offering attractive benefits in terms of ease of use and low cost [207–209].

In a recent study, the aim was to knock out the encoding genes *Anxa2*, which encodes annexin 2, and *Ctsd*, which encodes cathepsin D, using CRISPR-Cas9 to block the synthesis of these HCPs [204]. Meanwhile, Dovgan et al. (2021) [183] successfully minimised fragmentation levels in a drug product after knocking down cathepsin D through short hairpin RNA (shRNA) using CRISPR-Cas9. Similarly, Kol et al. (2020) [210] used multiplex CRISPR-Cas9 knock out multiple genes encoding problematic HCPs. In their study, they produced three types of clones: 6-protein knockouts, 11-protein knockouts, and 14-protein knockouts. Their analysis shows a significant reduction in HCP content, along with improved productivity and growth. Nonetheless, Laux et al. (2018) [206] removed the matripase-1 gene, a protease responsible for proteolytic degradation, using transcription activator-like effector nucleases (TALENs) and Zinc Finger Nucleases (ZFNs). It is also worth highlighting the study by Weiß et al. (2024) [205], which used artificial microRNAs (amiRNAs) to simultaneously knock down lipases with a single amiRNA, with potential for future genome editing.

These recent reports on eliminating specific proteins targets show positive results, with substantial reductions in HCPs and minimal degradation of recombinant proteins and polysorbate. These gene-reduction strategies do not affect cell growth or viability and can generate a superior CHO cell line. This effort will need some time to produce an ideal CHO cell line for biologics production [211]. Tuameh et al. (2023) [22] reviewed many upstream and downstream engineering-based methods for addressing HCPs in biologics product development, highlighting the importance of innovative emerging technologies. Recently, MS and surface plasmon resonance were used to investigate the role of lipase glycans in enhancing non-covalent HCP-mAb binding [212]. The study suggested manipulating HCP glycosylation by exploring feed conditions and developing a cell line with partially deglycosylated lipases through asparagine-mutagenesis or mutagenesis of the antibody CH1 domain.

### Protein A column improved utilisation and alternative treatments

Downstream processes remain crucial for achieving the levels of HCP clearance required for therapeutic use. The traditional practice of impurity removal begins during cell clarification. The clarification process typically uses methods such as centrifugation, depth filtration, and, less commonly in mammalian cell culture, precipitation. Single-use depth filtration has been widely implemented due to its ease of handling and cleaning. The sequence of the purification unit operation depends on the developed therapeutic molecules. Monoclonal antibodies or proteins containing the Fc fragment would typically be processed using a cost-intensive protein A affinity chromatography column to remove most HCPs [197].

However, the increasing mAb titre and cell densities in current upstream systems require innovative and cost-effective approaches for clarification and purification [213–215]. During clarification, high shear stress can cause HCPs to be released from cells [215, 216]. Figure 1 in the introduction (part 4) and Table 2 below show the most recent innovative downstream technologies for improving HCP removal at a lower cost. The studies include CHO cell flocculation experiments, attempts to replace the protein A column entirely, and efforts to enhance the purity of the feed to extend the protein A column’s lifetime. As some of these studies reported high purity and yield, it is important to consider the ratio of impurity to product, as shown in the third column of Table 2. These impurities in the proof-of-concept studies are usually HCP mixtures, but common proteins such as bovine serum albumin (BSA) were used for simplicity, while the product is often mAbs. Although most commercial cell culture media are serum-free nowadays, their interactions with mAbs differ from those of the HCP mixture.

**Table 2:** Some of the recent published downstream technologies to improve CHO HCPs removal and process economics chronologically. Activated carbon (AC), Antion exchange membrane (AEM), centrifugation (C), cation exchange chromatography (CEX), depth filtration (DF), flocculation (F), ionic liquid (IL), ultrafiltration (U), precipitation (P), microfiltration (MF), multimode chromatography (MMC), and tangential flow filtration (TFF)

Technology	Comment	HCPs/mAb^A^	Reference
Cationic F	Poly-L-amino acids and polyethylenimine were effective at removing CHO cells, HCP, and DNA. However, polyethylenimine affected EPO glycans but not mAbs	0.3/1 (mAb) 100(EPO)	[217]
P + CEX + MMC + AEM*	Achieved a 75% yield with acceptable product quality	0.18/1.8–5	[218]
P	Acidic salt precipitation of chromatin heteroaggregate improved impurities removal at capture step	0.44/1.31	[219]
Cationic F	Chitosan outperformed pDADMAC in clarification efficiency, with high DNA removal and mAb recovery	Not disclosed	[220]
IL P + UF*	Enabled mAb removal and IL reuse, reaching > 60% purity. A promising sustainable approach	0.332/0.0677	[221]
Modified UF membrane	UF at pH 9 achieved a maximum purity of 96%. A subsequent diafiltration step was suggested to increase yield beyond 46%	0.5/0.2	[222]
DF + MF + TFF + CEX*	Chromatin clarification by a fatty acid removed most DNA and histones, with 92.5% IgG recovery, meeting all clinical requirements	0.519–0.03/1.5	[196]
P + DF + UF + AC + AEM*	Designing a pilot-scale, column-free flow-through purification process with the potential to improve process economy and efficiency	0.25/4.6	[223]
Diatomaceous earth DF	Used alone or post-centrifugation; selectively removed positively charged hydrophobic proteins, but not those near their pI	0.2/Not disclosed	[213]
Peptide ligands*	2.7 HCP logarithmic removal value at constant yield and purity along 100 chromatographic cycles	0.22/1.12	[224]
Multiple filters	DNA reduction at the protein A load stage directly lowered HCP in eluate	1.2/0.8–2.9	[225]
P + F + C + DF	Achieved > 91% mAb recovery, low turbidity, and >91% DNA removal	0.3–0.5/3	[216]
Silica supports modified with IL*	Purification with 76% yield and 100% purity on support surface	0.2–0.53/0.05 -0.5	[226]
Chromatographic clarifiers	Reduced filter area and flushing volume; cost savings of 17–30%	500,000 (ppm) Not disclosed	[227]
DF	Compared three industrial DF trains; observed no retention of HCP/DNA with overloading	13–26/6.5	[214]
Hollow fibre membrane	Resulted in >95% mAb yield and >100-fold HCP reduction	5.2/5	[228]
Protein A membranes	Provided high HCP and DNA removal without affecting recovery. GORE™ and HiTrap Fibro™ showed good flow and binding	0.3/0.2–5	[229]

*Addressing the protein A replacement

**A** the feed concentration in mg mL^-1^ unless other unit stated, the HCPs might be BSA and others

To improve clarity, Agarwal et al. (2023) [230] suggested a single-use continuous-discharge-disk-stack centrifuge as an alternative, given its ability to process high cell density cultures. Another improved primary clarification method is a combination of a low-pH precipitation, flocculation, fluidised-bed centrifugation, and filtration [216]. Moreover, Liu et al. (2019) [219] suggested low pH treatment using acetic acid, which led to HCP precipitation. Similarly, Kateja et al. (2018) [218] used the precipitation method as the first step in their exploration of the non-protein A platform. Precipitation and immunoglobulin adsorption to porous zirconia particles were proposed as alternative to the protein A column, with high purity and good yield [231].

Aggregates should be specifically removed as they enhance HCPs’ persistence in the downstream train [196]. Furthermore, Gupta et al. (2020) [232] summarised the commercially available encapsulated adsorptive depth filters, particle-sieving nonwoven filters, melt-blown polypropylene, and glass microfiber from different vendors (i.e., MilliporeSigma, Pall, Sartorius, and 3 M). These filter membranes have been proven effective for use pre chromatography columns. Some of these brand names may now be different, as Pall Corporation is now part of Cytiva, and 3 M’s healthcare business is now a separate company known as Solventum Corporation. In another study, cholesterol concentration and particle size were used as indicator of clarification performance, while LDH was used as an indicator of cell lysis during shear studies [233].

Flocculating agents, specifically cationic polymers such as chitosan and polydiallyldimethylammonium chloride (PDADMAC), were suggested for removing negatively charged HCPs [220]. Furthermore, metal cations have been explored for HCP removal; for instance, calcium phosphate was reported to achieve high HCP removal [234]. A flocculation study concluded that poly-l-amino acids effectively removed cells, HCP, and DNA. At the same time, polyethylenimine (PEI) could negatively affect the glycosylated component of erythropoietin but not the examined mAb, even though it was the best among the tested flocculating agents, as shown in the first row of Table 2 [217]. Flocculation and membrane filtration for supernatant clarification were suggested by many authors [217, 220, 235]. More proposed cost-effective clarification approaches include recovery of mAbs through precipitation with ionic liquids, which alone cannot achieve high purity, as shown in Table 2 [221].

Following primary clarification, newer post-clarification processing strategies, such as chromatographic clarifiers, are now being explored as intermediate processing steps. The strategies reduce HCP levels, easing the load on downstream purification, particularly the protein A capture step [227, 228]. Using various media, these clarifiers incorporate an anion exchange membrane that specifically targets the removal of host cell DNA or chromatin [225]. By eliminating these impurities, HCP co-elution could be minimised in the subsequent protein A capture step. Chromatographic membrane design and future trends for mAbs purification have been extensively reviewed recently to improve protein purification throughput and process economics [236].

Among all downstream purification steps, the protein A capture step has the highest capital cost. This has led to increased interest in modified protein A ligands to extend the lifetime during alkaline washing (i.e., pH 10) and improve mAb yield [45]. Likewise, protein A ligand was engineered to tolerate up to 1.5 M NaoH cleaning-in-place (CIP) [237]. An imprinted mAb within a polyhydroxyethyl methacrylate monolith containing methacrylate as the functional monomer was used to selectively capture IgG from human plasma [238]. Similarly, a newly synthesised molecular imprinted thiophilic ligand linked to a peptide-crosslink was proposed as a cheaper, better alternative to protein A, achieving yield and purity of over 85% [239]. Although thiophilic ligands were discovered in 1985 and can capture mAbs at constant pH through the Fab domain, they have a limited number of publications compared to other ligands [240]. In addition, it needs to be followed by other chromatography column such as SEC to improve monomer content and achieve >95% purity [241].

Another proposed mAb capture step is phase partitioning, a method that separates biomolecules based on their tendency to partition between phases, depending on properties such as hydrophobicity and charge [242]. HCP from serum-free CHO cell culture supernatant was removed by partitioning in a polyethylene glycol-salt aqueous biphasic system, which introduces ionic liquids as adjuvants. It was discovered that supported ionic liquids may be tailored to eliminate contaminants or selectively capture mAbs without compromising the biological function of the antibody product. Another promising strategy to replace the protein A traditional resin and column is the use of protein A membrane adsorbers. The resin-based chromatography column has diffusion-limited mass transfer hence protein A chromatographic membranes are commercially available to increase productivity [243]. In a recent study, three commercially available membrane adsorbers demonstrated their capability to achieve high levels of HCP clearance [229]. Moreover, modification of a polyethersulfone membrane with polyethyleneimine, which retains negatively charged impurities from supernatant, achieved over 95% antibody purity and a yield of 46% [222].

Optimising pH pre-elution and elution conditions for the protein A column was shown to prevent aggregation and denaturation due to harsh pH [244]. This is important to reduce capture costs as protein stabiliser addition is expensive to utilise and remove after the protein A column. Moreover, Lin and Wang (2025) [244] suggested gentler pH shift and elution to reduce HCPs co-elution with mAbs, opening a new window for process optimisation. They also suggested solutions to the resulting larger elution volumes and lower product concentration in the eluted fractions, such as the implementation of tangential flow filtration after protein A capture. Among the various amino acid-based elution buffers tested, leucine, glycine, and serine provided the most effective pH transition. In a previous study, glycine buffer for acidic elution in protein A affinity chromatography provided better impurity removal than the more commonly used acetate buffer in the biopharmaceutical industry [245]. Greener mAb purification technology was published recently, that uses a wood backbone and a brush-like graft dextran layer coupled with a protein A column [246]. The authors admitted the need for improving the grafting process and further assessment regarding its long-term stability. This invention offers a sustainable, cost-effective, and efficient chromatographic matrix for mAbs purification. It is an early proof-of-concept study, as improving impurity removal was not its purpose.

Many researchers have been exploring the use of non-protein A platforms as an alternative means to reduce downstream processing costs. After sufficient clarification, the purification of mAbs can proceed directly to cation-exchange chromatography [218]. Activated carbon membrane combined with ultrafiltration/dilution was also proposed as a column-free downstream process [223]. The same authors also suggested integrated flow-through activated carbon process for post-protein A treatment [247]. In addition, biomimetic peptide affinity chromatography has been suggested as a capture step, achieving reasonable mAb recovery and low HCP levels after process optimisation [248].

In contrast to product capture and elution, targeted HCP removal from cell culture fluid was achieved using peptide-based LigaGuard™ resins that continuously target CHO HCPs [249]. It was shown that most high-risk HCPs (e.g., histones, lipoprotein lipases, cathepsins, and glutathione S-transferase) were removed with high mAb yield. It is worth noting that the HCP capture step was proposed before or after the protein A column, although the examined feed in the study contains much less HCP/mAb than the culture fluid of a CHO fed-batch culture. This invention has potential for protein A-free mAb manufacturing and viral vector production.

Changing surface hydrophobicity and charge distribution between HCPs and the target protein form the basis for separation strategies using mixed-mode resins. Resins such as Capto Adhere, and Capto MMC, have demonstrated significant improvements in HCP removal when applied in bind-elute and/or flow-through purification modes [46]. Conjugated MMC outperformed Capto MMC during mAb capture in binding capacity, HCPs, and aggregates removal [250]. Resin development remains an attractive topic to enhance HCP removal. An innovative simultaneous clarifying method for CHO cell supernatant and Trastuzumab capture was recently described, using calcium-dependent magnetic separation [251]. Product recovery above 90% was achieved at pH 6.0 without compromising product integrity or binding capacity. These innovations include resins with tetrameric and hexameric ligands composed of multipolar or hydrophobic/positively charged amino acids, as well as those incorporating hydrophobically modified polyallylamine (PAA-butyl) and 2-amino-4-methylpentanoic acid ligands.

These technologies have proven highly effective in reducing both high- and low-molecular-weight HCPs. It is crucial for process understanding, validation, and optimisation to know whether the supernatant has been diluted or concentrated before choosing the right technology to capture or polish mAbs. Although some technologies achieve high yields and purities, studies often use different impurity-to-product ratios and simpler HCP mixtures, that do not always reflect industrially relevant conditions, hindering direct comparisons between studies. These emerging technologies would require further development to meet Good Manufacturing Practice (GMP) requirements.

## In silico approaches for comprehensive HCP assessmenti

### Process mathematical models and optimisation

There are a few mathematical tools to understand the dynamics of HCP in upstream and downstream unit operations [252, 253]. The upstream mathematical models are used to model the process, with an objective function that maximises titre and purity by controlling nutrient feed, temperature downshift, and culture duration. An example of such bioreactor models was provided by Alhuthali and Kontoravdi (2022) [14], who used population balance modeling to maximise the mAb-to-HCP ratio. The model highlights the importance of accounting for the CHO cell HCP active secretion and degradation during cell culture. The study recommended fresh sample analysis when quantifying DNA, LDH, and HCP for cell lysis. Another model given by Okamura et al., incorporated both DNA and HCP into a CHO cell culture mathematical model [254].

A downstream example of HCP modelling potential is the chromatography mathematical model, which account for HCP impurities [255]. The model evaluates the impact of loading residence time on HCPs and aggregates for optimisation purposes. Recently, a mechanistic model for protein A chromatography was proposed, demonstrating good predictive capability and suitable for larger columns [256]. In another study, column loading flow rate was optimised by multi-objective optimisation to increase productivity and resin utilisation [257].

A recent method was developed to determine the binding parameters of individual HCPs in an *E. coli* harvest sample [258]. The proposed model accurately predicts the independently measured retention times of 15 HCPs during the validation gradient run. The anion exchange chromatography capture step was optimised in silico, paving the way for more detailed HCP removal optimisation. Likewise, the 3D structures of many *E. coli* HCPs were recently integrated into a chromatography model to understand the elution behaviour of each HCP, thereby improving the design of purification processes [139]. Cross validation R^2^ of around 0.7 was obtained, and further research in this direction is needed to improve model performance.

### Genome scale model to guide genetic modification

Systems biology models such as genome-scale models, are used to assess metabolic engineering interventions such as gene knockout for problematic or immunogenic HCP. These models are the result of genome sequencing efforts to construct metabolic networks for different eukaryotic and prokaryotic cells for medicine and industry [259, 260]. It is known that the protein secretory pathway can be genetically engineered to suit better the needs of the biopharmaceutical industry [210]. However, as emphasised in Sect. “HCP quantification progress and challenges”, accurate detection, quantification, and characterisation of HCPs are crucial for prioritising these genetic interventions, without sacrificing cell growth and therapeutic product quality.

An example of this strategy is given by Kol et al. (2020) [210], who used a genome-scale metabolic model of CHO cells to describe the protein secretory pathway and identify non-essential HCPs that incur a high energy cost for their synthesis and secretion. The results informed a series of sequential knock-out experiments to create a leaner cell line that provided a cleaner feedstock to downstream purification. It is essential to ensure that these HCPs do not affect cellular growth or the therapeutic protein’s quality attributes, such as glycosylation, before gene deletion.

### HCP immunogenicity prediction

The FDA’s 2014 Guidance for Industry recommends that manufacturers monitor subvisible particles in the 2 to 10 μm range throughout a product’s shelf life and characterise particles between 0.1 and 2 microns. Notably, studies have shown that subvisible aggregates ranging from 0.1 to 100 microns can significantly increase immunogenicity [261]. Moreover, subvisible particles composed of free fatty acids resulting from polysorbate hydrolysis are usually observed within a year storage at 2–8°C. Reducing PLBL-2 to LOD 0.6 results in no subvisible particles for 2 years of storage. A revised manufacturing process for abicipar was developed to reduce *E. coli* HCPs, resulting in a moderately lower incidence and severity of adverse effects [262]. These studies highlight the importance of continuous HCP detection and removal strategies throughout the therapeutic product life cycle to extend its shelf life, and they emphasise the relationship between HCPs and aggregate formation.

Computational tools are used to assess HCP immunogenicity from the HCP amino acid sequence to T cell activation prediction [263]. This type of model was initially used for vaccine design to trigger some immune response in humans [264, 265]. The immunogenic response tools use epitope prediction models, such as TepiTool and NetMHCIIpan, as well as immunoinformatic databases, such as the Immune Epitope Database (IEDB) [266–268]. It is worth mentioning that TepiTool uses a combination of machine learning models, whereas NetMHCIIpan uses artificial neural networks. These publicly available in silico tools have been used for therapeutic protein design, such as mAbs to prevent the formation of anti-drug antibody after injection [269]. An accurate immunogenicity prediction tool would attract a lot of industrial attention and regulatory bodies, as it has the potential to reduce the number of participants needed for clinical trials [270].

The use of in silico assessment of HCP immunogenicity was initially suggested by members of the EpiVax company, who developed the online tool CHOPPI to prioritise the removal of immunogenic HCPs [271]. The same group recently suggested using human T cell response prediction and in vitro validation methods to assess CHO HCPs in biopharmaceutical development [24, 272]. These methods are summarised in the review structure, Fig. 1 (part 5 in the introduction). Moreover, in collaboration with the FDA, the EpiVax approach was recently used to evaluate the immunogenicity of salmon calcitonin peptide HCP impurities, which were then validated by Class II human leukocyte antigen (HLA)-binding and T-cell assays [273].

The immunogenicity assessment software provides an immunogenicity prediction score based on multiple computer algorithms developed to screen for putative epitopes. An example is the commercial EpiMatrix, which predicts immunogenicity based on the affinity of a single amino acid to one of the nine positions within the binding groove of an HLA molecule (i.e., common Class II HLA-DR supertype alleles selected for >95% global population coverage). Although there are many other algorithms, EpiMatrix is recommended for assessing HCPs’ immunogenicity due to its high accuracy and validation in vitro and in vivo studies, according to the developers [274]. The EpiMatrix algorithm utilises Class I and Class II HLA “pocket profiles” which represent HLA pocket binding coefficients and employs these coefficients to predict overlapping 9- and 10-mer peptide epitopes. EpiMatrix evaluates whole protein immunogenicity by summing the top 5% of predicted binder scores across common HLA alleles. It normalises this value to a 1000-amino-acid protein length and compares it to a random baseline. The resulting score ranges from −50 to +50, with zero representing random expectation. A score above zero suggests an increased number of MHC (Major Histocompatibility Complex) ligands and higher immunogenicity potential, while a score below zero indicates fewer ligands and lower immunogenicity. Proteins scoring above 20 are considered significantly immunogenic.

Moreover, normalisation against the mean score of a random set of peptides, and deviations from that mean are reported as Z-scores. Z-scores indicate the potential of each 9-mer frame to bind to a given HLA allele supertype. A peptide from any sequence is likely to bind to HLA and be presented on the surface of antigen-presenting cells if Z-score is statistically significant (i.e., higher than 1.64). More about the interpretation of the data can be found in the supplemental information of De Groot et al. (2023) [275]. The ClustiMer tool is usually used to screen longer sequences of up to 45 amino acids to identify high-T-cell epitope regions, as they tend to be clustered [24].

Identifying HLA-DR binding epitopes alone is not sufficient to assess the immunogenicity of peptides in therapeutic proteins or HCPs. A key reason is that HCP epitopes from mammalian cell lines such as CHO or HEK cells may closely resemble human genome epitopes that the immune system tolerates. To address this, an additional step in the EpiMatrix result is needed (i.e., the JanusMatrix tool) to analyse the similarity between HLA-binding T cell epitope clusters in HCPs and their human proteome counterparts. JanusMatrix extends the sequence alignment search beyond Basic Local Alignment Search Tool (BLAST) against the human genome to eliminate epitopes that could be considered as self [276]. Cross-reactivity between HCP epitopes and other self-epitopes can shift to other similar epitopes in the host body or the administered therapeutic proteins [271]. This approach was recently suggested by EpiVax’s scientists as integrated algorithms for immunogenicity screening of HCPs, known as ISPRI-HCP [24]. ISPRI-HCP was used to determine the immunogenic potential of the 143 CHO HCPs that often co-purify with mAbs. It was also used to assess adenoviral vector-derived impurity in the AstraZeneca nCoV-19 vaccine. Mattei et al. (2024) [272] recently used ISPRI to suggest protein sequence modifications to moderate putative T-cell epitopes in some therapeutic proteins. A lack of user-friendly interfaces and instructions often limits the popularity of these models. Because of the inherent heterogeneity of the immune system and the uncertainty in the algorithms used, these in silico tools need to be combined to guide experimental design for HCP immunogenicity based on foreign epitopes and HCP concentration.

### In vitro HCP immunogenicity assessment and validation

Nonclinical immunogenicity assessment tools are valuable for evaluating new therapeutic candidates and mutations, as demonstrated by the recent case of the knobs-into-holes bispecific antibody, which was deemed safe based on both in silico and in vitro results [277]. Computer-based immunogenicity results may be verified by in vitro T-cell activation and cytokine secretion assays, using human donors’ peripheral blood mononuclear cells (PBMCs) [274]. Jawa et al. (2016) [278] conducted one of the early studies using a PMBC-based assay in parallel to an immunogenicity prediction tool to evaluate over 20 common CHO HCPs. It was reported that HCP concentrations up to 40 times the 100 ppm threshold did not trigger higher immunogenicity in an in vitro assay, although in-silico prediction classified a few HCPs as immunogenic. These discrepancies between in silico and in vitro validation prompted further investigations, as discussed in this section. They highlight the role of HCP concentration in triggering immune responses, as well as the need for multiple complementary assessment approaches to achieve a comprehensive evaluation of HCP immunogenicity.

Binding affinity assays (i.e., MHC-peptide interactions) are also used to measure MHC-peptide binding directly, but binding alone does not guarantee T-cell activation. The first in vitro validation of six problematic CHO HCPs spiked into Rituximab was recently published, using antigen-presenting cells, namely dendritic cells. HLA-DR and CD86 were measured as activation markers for commonly found CHO HCPs (PRDX1, S100A4, PLBL2, CCL2, CLU, and YWHAE) with different purification processes [279]. The maturation markers were downregulated when Rituximab was present, although the CD86 signal, which indicates adjuvant activity, was positively correlated with three known immunogenic HCPs (i.e., PRDX1, S100A4, and PLBL2) when Rituximab was absent. CD86 is known to upregulate more rapidly than HA-DR, providing immediate support for T cell activation. The study’s primary purpose was to assess the adjuvanticity effect rather than to conduct a thorough immunogenicity assessment of these HCPs to validate a previous in silico result.

In vitro binding and cell-based immunogenicity assessment assays have been extensively reviewed in the literature for therapeutic proteins spiked with foreign proteins or peptides [280]. The review highlights important factors regarding patients and tested products to design the most appropriate experiment. These factors are assay type (i.e., mainly DC-T cells from PBMCs), donor state, number of in vitro immune cells, proteinaceous product, stimulation protein, cell culture duration, and readout variables. It is advisable to select a diverse donor population with a range of HLA alleles. Different MHC class II alleles and subtypes vary in their ability to bind antigen-derived peptides, meaning that individual variations in MHC protein sequences can affect immune response development. Notably, particular MHC class II alleles are associated with increased susceptibility to specific diseases and variations in immune reactivity. However, if the test is conducted in a specific disease or population, it is recommended to narrow in silico or in vitro testing to focus on this patient population.

There is concern that monocyte-derived dendritic cell (moDC)-T cells from healthy donors may not necessarily represent the immune reflect of the patient population. Moreover, PBMC from the same healthy donors have heterogeneity in the ratio of antigen-presenting cells to T cells. Hence a more labour-intensive approach, such as CD4+ T-enriched PBMCs, is used to increase sensitivity and throughput [25, 281]. Most of this immunogenicity is directed toward the therapeutic protein, assessed through anti-drug antibodies identification rather than T cell activation in response to product impurities.

A detailed protocol for major histocompatibility complex-II (MHC-II)-Associated Peptide Proteomic (MAPPs) was reported in the literature, with an extensive review of standard protocols for drug-induced immunogenicity, which uses LC–MS/MS to analyse mature moDC lysate [282, 283]. Most of these assays start from PBMCs isolation from healthy donors, followed by cell type separation and culture (e.g., immature DCs differentiated from monocytes). The differentiation period varies, but 5 days are usually sufficient to differentiate CD14+ cells into moDCs [284]. The panel of activation examined markers in the study are HLA-DR, CD40, CD86, CD83, CD209, and CD80. Process-related impurities and the therapeutic protein immune response have been assessed using these assays, including AAV empty capsids and Cas9 protein for cell therapy applications [59]. Therefore, based on a cynomolgus macaque study, assessing the magnitude and frequency of pre-existing immune responses, along with considering MHC class II genetics, has been proposed as a valuable approach for evaluating the immunogenic potential of biotherapeutics [285].

Recently, the different PBMCs subgroups (CD8 T cell-depleted PBMC, co-culture dendritic cells (DCs), and autologous CD4 T cells) were assessed for immunogenicity using 10 diverse mAbs across different investigation settings, including restimulation with monocytes [286]. It was reported that three mAbs did not elicit an immune response in these assays, despite being known to be highly immunogenic. It was concluded that there is no one-size-fits-all CD4 T assay; instead, multiple assays and tools should be used to assess and minimise immunogenicity risk. Recently, ninety immune-related genes were monitored in a comparative study to evaluate the immunogenicity of a biosimilar insulin [270]. The study suggested that products expressed in *E. coli* and *Komagataella pastoris* trigger similar innate immune responses, highlighting the value of these assays.

Panikulam et al. (2025) [25] reported several in vitro and in vivo studies assessing adjuvanticity and immunogenicity risks of HCPs derived from *E. coli* and mammalian cell lines. Patient-related factors, such as age and disease state, and treatment-related factors, such as route of administration and dosing frequency, can influence the immune risk associated with HCPs. Yasuno et al. (2020) assessed the immunogenicity of *E. coli* HCPs at up to 10 ppm integrated analyses in vitro differentiated THP-1 cells and an in vivo rabbit model [287]. Cytokines and cell surface markers (CD54, CD80, and CD86) were measured. It was concluded that HCPs might induce inflammation and immunogenicity as an adjuvant.

Although the post-translational modifications of individual HCPs have not explicitly been studied, research on biotherapeutics has shown that non-human glycans (e.g., galactose-α-1,3-galactose, N-glycolylneuraminic acid) and glycan linkages (e.g., α1–3-galactose, α1–3-fucose) can be highly immunogenic. The authors of these reviews agree that there is no current consensus on the most sensitive or preferred approach, but that a combination of tests is beneficial for measuring a drug’s immunogenicity. These studies require skilled technicians and advanced facilities to maintain cell culture for a few days. It also needs process optimisation to improve accuracy and reproducibility [288]. One issue with in vitro assays is that the immune response varies among patients and is likely more sensitive than the assays, as supported by a Ranibizumab study with low HCPs [25].

## HCP challenges in biosimilars and next generation biologics

The cost of a biosimilar product is expected to be 30% less than the reference biologic, with strict guidelines for therapeutic evaluation and post-marketing surveillance [289]. Some innovative therapeutic proteins have received regulatory approval for commercialisation, yet their purification processes and formulations are less frequently discussed due to intellectual property barriers [290]. One industrial challenge is that the HCP profile of a proposed biosimilar is not expected to match the reference product, as it depends on the cell line and purification process design [291]. New modalities not covered in this review include covalently modified proteins (e.g., PEGylated, albumin-fused, Fc-fused, or lipidated); PEGylated and lipidated forms often requiring an additional purification step [292].

Modifications in cell line/bank, dosage form, formulation, and production site have been witnessed in the literature to improve process economics [293]. These changes must be well understood and thoroughly assessed to maintain regulatory approval, supported by comprehensive clinical similarity studies that include HCP analysis. An example of this is a three-way analytical comparability study following a change from a low-titre CHO cell line to a high-titre CHO cell line expressing a bevacizumab biosimilar [294]. Sharma et al. (2026) [295] recently reviewed the manufacturing differences between aflibercept and ranibizumab biosimilars, focusing on cell lines and purification processes, but without including any details about HCPs. It was mentioned that aflibercept is produced using CHO system, whereas ranibizumab is expressed as inclusion bodies in *E. coli*.

Molecular modifications of biologics strongly affect the purification strategies and related cost. Sanchez-Trasvina et al. (2021) [292] gave the example of coagulation factor IX, the only therapeutic protein on the market with three different modifications that affect its purification approach. These purification concerns have been confirmed by Chu et al. (2021) [296], who published a comprehensive review about peptide and pseudopeptide-based ligand design to overcome challenges in the purification of next-generation biotherapeutics. Comprehensive comparative studies of HCPs in biosimilar and reference products are scarce in the literature. However, as more biosimilars become commercially available, academic research is expected to increasingly investigate similarities in purification processes, as well as process- and product-related impurity profiles.

### Multispecific antibodies and mAb fragments

As therapeutic proteins become more complex, standard downstream processes may need to be modified to reduce product-related impurities and HCP co-purification, particularly for bispecific and Fab-based products [132, 297, 298]. Some of these mAb fragments miss the Fc region; hence, an alternative to protein A chromatography is needed. For example, Eloctate® and Nplate® are two Fc-fusion proteins that do not utilise a protein A affinity chromatography column, as the former uses an affinity nanobody ligand and the latter is isolated from inclusion bodies [292, 299]. A wide range of ligands exhibit affinity for nearly all parts of mAbs. For instance, CaptureSelect FcXP and MabSelect VH3 target the VH3 region of the heavy chain [299, 300]; CaptureSelect CH1-XL binds the CH1 domain [301]; protein A and protein G bind the CH2/CH3 domains of the Fc region [299]; Capto L (i.e., similar to protein L affinity chromatography) targets the variable region of the kappa light chain [297, 302]; KappaSelect binds the constant region of the kappa light chain; and LambdaFabSelect targets the constant region of the lambda light chain. It is worth noting that ligand names may vary among vendors and that products may be discontinued; however, identifying the region of the therapeutic protein used to capture it the affinity chromatography is critical, as it represents the primary criterion for purifying multispecific antibodies from product-related impurities [302]. When therapeutic proteins are very sensitive to low pH, other affinity chromatography methods or MMC may be used [46, 292]. However, acidic treatment used to inactivate viral contamination is crucial in therapeutic protein production, and various methods are employed to prevent aggregation [303, 304].

Publication about purification of bispecific antibodies focuses currently on the removal of fragments, homodimers and aggregates more than on HCPs by commercially available protein A resins (e.g., dual binding capabilities to the Fc and VH3 regions) [299, 302]. This is because these modalities tend to produce more product-related impurities. However, with the new chromatographic ligands developed for these diverse molecules, their efficiency of removing HCP will be expected in the coming years when many move towards clinical assessment phases. Ceramic hydroxyapatite (CHA) with salt addition has been suggested as a polishing step to remove both process- and product- related impurities from a bispecific antibody although protein A column gave the highest reduction percentage regarding all classes of impurities for all three investigated bispecific molecules [305].

Binding domain composition analysis and process optimisation of a bispecific antibody (i.e., Faricimab) have been used to choose the best out of four commercially available ligands to increase purity to above 70% from 30% with 86% monomeric yield, including the partial removal of HCPs and DNA [302]. Similarly, four commercially available affinity resins (protein A and three non-protein A resins) were assessed for trispecific antibody purification from half antibodies, homodimers and aggregates [306]. Amino acid mutations in the constant light chain of a multi-specific antibody (i.e., crossover dual variable domain) were conducted to abolish the binding to the affinity chromatography ligand, removing product-related impurities [307].

With advances in antibody engineering and metabolic engineering, bispecific and trispecific antibodies are expected to enhance therapeutic efficacy, especially against tumours, through T cell receptor [308]. Recently, a trispecific antibody was purified by protein A column, followed by dialysis against PBS overnight and size-exclusion chromatography [308]. Many of these are still in the research and development phase; therefore, further purification and scale-up studies focusing on impurity removal are expected to appear in the literature.

Wang et al. (2024) [298] highlighted the accumulation of byproducts, including high- and low- MW variants and homodimers, during bispecific antibody production. They successfully designed a non-protein A process comprising two chromatography columns (i.e., MMC capture step followed by AEX), achieving 98% impurity removal with controlled HCP levels at 10 ppm and 60% recovery. An MMC featuring weak cation exchange and moderate hydrophobic interactions was successfully used to remove impurities from a highly hydrophobic bispecific antibody with a complex format, achieving 97% purity and 70% recovery in a single step [309]. These new affinity chromatography columns would interact differently with HCPs, potentially co-eluting different HCPs.

### Vaccines and other therapeutics

The ProteoMiner bead technology described in Sect. “Recent industry-related HCP LC–MS studies” was also applied recently to detect and quantify HCPs in an adeno-associated virus (AAV) gene therapy product [310]. This approach removes the surfactant Pluronic F-68 without compromising low-abundance HCPs, enabling detection at 0.1 ng/mL and demonstrating applicability to other gene therapy products. Two commercially available 0.22 microfilters were assessed in the study to remove HCPs and DNA from therapeutic virus (i.e., vesicular stomatitis virus) [311]. An increase in HCPs from 1 to 25 ug/mL resulted in significant membrane fouling, reducing virus recovery to as low as 48%. It is worth mentioning that bovine serum albumin did not cause the same issue, highlighting the fouling of the different behaviour of the HCPs mixture in these sterile filtrations’ performance. In a recent study, various lots of SARS-CoV-2 vaccines from AstraZeneca and Johnson & Johnson were analysed to screen for process- and product-related impurities. These two vaccines were produced in mammalian cells, and the HCP content in one exceeded the EMA’s 400 ng per dose threshold [157]. This type of academic scrutiny is rare in the literature, as it is typically analysed by the manufacturer, which puts greater pressure on the purity of future biologics.

Extracellular vesicles and exosomes are rapidly emerging therapeutic and drug-delivery platforms that utilise chromatography columns such as heparin affinity chromatography. Calnexin and human serum albumin were the two prominent residual proteins successfully removed with DNA by heparin affinity chromatography, achieving 98 and 99% removal, respectively [312]. Moreover, although no bacteriophage products have yet received full FDA approval for general human use, several clinical trials are underway to evaluate their safety and efficacy. Phages can be separated from the supernatant and small, free impurities such as HCPs using semi-permeable membranes. These membranes have pore sizes that allow impurities to pass through while retaining the larger phage particles in the retentate [313]. A recent metabolomic study reported the development of a new mammalian cell line to accelerate biologics production, using a Chinese hamster lung cell line to produce mAbs [314]. The newly developed cell line has a higher growth rate and greater metabolic demand than the CHO cell line. HCP dynamics in these cell lines might differ, so a new list of problematic HCPs might emerge.

## Conclusion

The detection and quantification of HCPs have improved over the past few years with the development of innovative approaches, such as therapeutic product removal, HCP enrichment and fractionation, the addition of surfactants during digestion, alternative digestion workflows, better calibration curve standards, longer chromatographic columns, nanoliter flowrates, and advanced MS instrumentation and data acquisition methods. MS-based approaches are increasingly central to modern biopharmaceutical quality control when integrated with risk-based analytical strategies. Despite the known underestimation by ELISA, commercially available and in-house ELISAs developed for a specific therapeutic product and process remain valuable for early process development and rapid global HCP measurement.

The list of difficult-to-remove HCPs depend on the therapeutic product, and manufacturing process. The list of high-risk HCPs depends on the cell line and our understanding of HCP species degradation potential and immunogenicity. Protein A chromatography currently has no cheaper, equally efficient alternative that has gained wide acceptance for therapeutics containing the Fc region. However, process development and innovation in mAb purification may extend its lifetime and improve its utilisation as a clarification step preceding mAb capture. Although combinations of alternative methods used in sequence could potentially replace protein A chromatography, they would increase process complexity. However, such approaches may improve the economics of therapeutic protein processing by reducing purification costs and enabling the evaluation of synergistic effects among emerging technologies for mAb purification. Before integration into a GMP platform, these purification technologies must prove their stability and effectiveness against a range of process- and product- related impurities.

Multifaceted assessment approaches for HCP measurements and immunogenicity (i.e., a combination of in silico and in vitro methods) are required, as there is no single, comprehensive, easy, quick, and cost-effective platform. The success of a biosimilar company will depend in part on how quickly it adopts new technologies to cut manufacturing costs and stay competitive. More effort is needed to drive innovation in HCP removal and reduce purification costs for new therapeutic proteins and biosimilars.

## Data Availability

No datasets were generated or analysed during the current study.

## Abbreviations

AAV: Adeno-associated virus
ACN: Acetonitrile
amiRNA: Artificial microRNA
AEX: Anion exchange chromatography
BLAST: Basic local alignment search tool
BSA: Bovine serum albumin
CE: Capillary electrophoresis
CHO: Chinese hamster ovary
CSH: Charged-surface hybrid
DC: Dendritic cell
DDA: Data-dependent acquisition
DIA: Data-independent acquisition
DNA: Deoxyribonucleotide acid
DtR: Difficult-to-remove
ELISA: Enzyme-linked immunosorbent assay
EMA: European medicines agency
FAIMS: Field asymmetric ion mobility spectrometry
FDA: Food and drug administration
FDR: False-discovery rates
GMP: Good manufacturing practice
GPF: Gas-phase fractionation
GPS: Gas-phase separation
HBsAg: Hepatitis B surface antigen
HCCF: Harvested cell culture fluid
HCP: Host cell proteins
HEK: Human embryonic kidney
HLA: Human leukocyte antigen
HRP: Horseradish peroxidase
ICH: International council for harmonisation
IgG: Immunoglobulin
IMAC: Immobilised metal affinity chromatography
kDa: Kilo dalton
LC-MS: Liquid chromatography and mass spectrometry
LDH: Lactate dehydrogenase
LPL: Lipoprotein lipase
mAb: Monoclonal antibody
MCP-1: Monocyte chemoattractant protein-1
MHC: Major histocompatibility complex
MMC: Multimodal chromatography
moDC: Monocyte-derived dendritic cell
MRM: Multiple reaction monitoring
MS: Mass spectrophotometry
mw: Molecular weight
NaCl: Sodium chloride
NIST: National institute of standards and technology
NS: Null strain
PBMC: Peripheral blood mononuclear cell
PDADMAC: Polydiallyldimethylammonium chloride
PEI: Polyethylenimine
pI: Isoelectric point
PLBL2: Phospholipase B-like 2
ppm: Parts per million
PRDX1: Peroxiredoxin-1
PRM: Parallel reaction monitoring
Q-TOF: Quadrupole time-of-flight
RM: Reference materials
RPLC: Reversed-phase liquid chromatography
RuBisCO: Ribulose-1,5-bisphosphate carboxylase/oxygenase)
SAAP: Surfactant-assisted acid precipitation
ScFv: Single-chain variable fragment
SDC: Sodium deoxycholate
SEC: Size-exclusion chromatography
shRNA: Short hairpin RNA
SIAE: Sialate O-acetyl esterase
SLS: Sodium lauroyl sarcosinate
SRM: Selected reaction monitoring
STD: Standard
SWATH: Sequential window acquisition of all theoretical fragment-ion spectra
TALENs: Transcription activator-like effector nucleases
TGF-β1: Transforming growth factor-beta 1
TLR5: Toll-like receptor 5
TMT: Tandem mass tag
UPR: Unfolded protein response
USP: United states pharmacopeia
ZFNs: Zinc finger nucleases
2D-DIGE: Two-dimensional difference in-gel electrophoresis
2D-PAGE: Two-dimensional polyacrylamide gel electrophoresis
2D-WB: 2D Western blot
